# Interventions for children with developmental coordination disorder: A scoping review

**DOI:** 10.1111/dmcn.70221

**Published:** 2026-02-26

**Authors:** Dorothee Jelsma, Zdenek Svoboda, Reza Abdollahipour, Miriam Palomo Nieto, Miguel Villa De Gregorio, Kamila Banatova, Farhad Ghadiri, Fatemeh Alaei, Paola Violasdotter Nilsson, Dido Green

**Affiliations:** ^1^ Clinical and Developmental Neuropsychology University of Groningen Groningen Netherlands; ^2^ Department of Natural Sciences in Kinanthropology, Faculty of Physical Culture Palacký University Olomouc Olomouc Czechia; ^3^ Department of Didactics of Languages, Arts and Physical Education, Faculty of Education‐Teacher Training Centre Complutense University of Madrid Madrid Spain; ^4^ Department of Motor Behavior, Faculty of Physical Education & Sport Sciences Kharazmi University Tehran Iran; ^5^ University Library Jönköping University Jönköping Sweden; ^6^ Department of Rehabilitation Jönköping University Jönköping Sweden

## Abstract

**Aim:**

To identify, qualify, and synthesize the characteristics of interventions for children with diagnosed and suspected developmental coordination disorder (DCD) worldwide.

**Method:**

This was a scoping review, with searches in eight bibliographical databases without language restrictions. All interventions involving children and adolescents with motor coordination disorders were included, with content analyses involving the type and context of the intervention and interventionists, the outcomes addressed, and the findings reported and considered regarding the International Classification of Functioning, Disability and Health framework.

**Results:**

From 30 637 hits, 202 distinct studies were identified, including 206 interventions with a total of 4593 children or adolescents. The children included were often poorly defined. Movement activities were offered through task‐oriented, motor skill, and sports training approaches with active video games and sensory‐perceptual motor skills training reported the most frequently. Long‐term psychosocial and participation outcomes were less explored.

**Interpretation:**

Future intervention studies need to be clearer on the diagnostic criteria and details of DCD, including individual(s) who delivered the training, the type of training, session intensity, and the delivery mode. Outcome measures should consider three critical domains: (1) practical application of learned motor skills in daily life; (2) environmental and psychosocial impacts; and (3) parental awareness and support.

## Graphical abstract

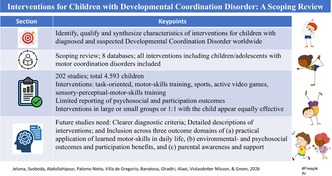

This scoping review identified 13 differing categories of interventions delivered to children with developmental coordination disorder worldwide ranging from task oriented, motor skills training, and sensorimotor approaches to sports training approaches and health promotion, with motor performance mostly reported as outcome and similar effectiveness whether delivered 1:1 or in (small) groups.

**Abbreviations**
DCD: developmental coordination disorder
ICF: International Classification of Functioning, Disability and Health
sDCD: suspected developmental coordination disorder

**What this paper adds**

- Thirteen categories of interventions were identified ranging from task‐oriented, motor skills training and sensorimotor approaches to sports training approaches.
- Most studies reported motor performance (acquiring and executing motor skills in daily activities) as an outcome measure.
- Interventions delivered in small or large groups or 1:1 showed similar reported improvements across differing contexts.
- Long‐term psychosocial outcomes and sustained participation benefits remain less explored.

Developmental coordination disorder (DCD) affects approximately 2% to 5% of children and is characterized by significant difficulties in acquiring and executing motor skills in daily activities (referred to here as ‘motor performance’).^1^, ^2^ Core features of DCD include delayed and less effective movement performance, impaired predictive motor control, and deficits in sensorimotor coordination.^3^, ^4^ Evidence suggests that these motor difficulties are persistent, with nearly half of children diagnosed in early childhood continuing to experience challenges into adolescence and early adulthood.^5^

Interventions for DCD encompass diverse approaches and include motor‐activity‐oriented interventions, active video gaming, and forms of cognitive training.^3^, ^4^ The 2019 international clinical practice guidelines on DCD intervention recommended activity‐oriented and participation‐oriented approaches to enhance general (overall movement competence and coordination), fundamental (core movement patterns such as balance, locomotion, and object manipulation), and specific motor skills,^3^ whereas Dannemiller et al.^4^ recommended task‐oriented interventions based on strong evidence, while interventions targeting body functions and structures (e.g. core stability, cardiorespiratory fitness, or power training) showed moderate effects. Smits‐Engelsman et al.^6^ further demonstrated that interventions closely aligned with tasks relevant to the child's everyday life yielded the greatest benefits. Although intervention studies are limited in number, they demonstrated significant variability in methods, delivery contexts, dosage, and duration.^3^

There is a lack of understanding regarding the types of treatment offered to children with DCD globally, limiting cross‐cultural information on motor proficiency, physical activity, and overall health outcomes.^7^ While fitness and function have been more common measures of motor capacity, a shift of focus to broader outcomes, also described as the 'F‐words' for rehabilitation, highlights psychosocial dimensions, including fun, friendship, family, and future.^8^ Lack of standardization in how and what outcomes are assessed—whether focusing on motor skills, participation, or broader quality of life indicators—complicates comparisons across studies and hinders the development of a unified approach to treatment. While several approaches have been developed for children with DCD, a comprehensive overview of interventions is lacking. Most reviews are systematic reviews that solely include randomized controlled trials and do not present a worldwide overview of interventions offered to individuals with DCD.

The aim of this scoping review was to identify, qualify, and synthesize the characteristics of interventions used for individuals with or at risk of DCD to also capture emerging areas of practice not included in recent reviews.^6^ The primary question was to describe what interventions were being used for children and adolescents with DCD since group‐based interventions were introduced.^3^ Secondary questions considered how these interventions were being implemented (including delivery methods, contexts, and dosage) and what outcomes were described.

## METHOD

A scoping review methodology was selected to capture the broad nature and context of currently implemented interventions across multiple contexts and cultures.^9^ This review followed the framework outlined by Arksey and O′Malley^10^ and Peters et al.^11^ according to five stages (question, identification, selection, charting, summarization); consultation was done within the BIG IDEAS for DCD intervention subgroup and results were reported according to the PRISMA Extension for Scoping Reviews guidelines.^12^ The protocol was registered with the International Platform of Registered Systematic Review and Meta‐Analysis Protocols (INPLASY202320028) on 7th February 2023 and last updated on 10th February 2023. As this review synthesized published and grey literature, ethical approval was not required.

### Eligibility criteria

We considered publications including children and young people aged 3 to 25 years, with disorders of motor coordination, DCD, suspected DCD (sDCD), or dyspraxia (see Table S1). Children with medical or neurological conditions, such as cerebral palsy or muscular dystrophy, were excluded. Studies, without language or study design restrictions, were included if they contained information on the type of intervention, the mode of delivery of interventions, the details of the interventionists, the context, the dosage and outcomes, and if published after 2011. Literature reviews were used to cross‐check references but were not otherwise included. Grey literature of non‐peer‐reviewed publications, and MSc and PhD theses, were included providing that the full text was accessible and data were not reported in other publications. If multiple publications reported on the same participants, these were registered as one study, but several outcomes were identified separately. Conference proceedings, abstracts, and posters were excluded if the full‐text publication could not be obtained.

### Search strategy

Working in conjunction with an experienced librarian (PVN) studies were identified by searching eight bibliographical databases: Allied and Complementary Medicine Database, APA PsycInfo, CINAHL with Full Text, Education Database, ERIC (searched via Education Collection), MEDLINE, Scopus, and Web of Science Core Collection (including the Science Citation Index‐EXPANDED, the Social Sciences Citation Index, the Arts and Humanities Citation Index, and the Emerging Sources Citation Index) and undertaken from the 13th to the 16th February 2023 and updated on 21st February 2024 and 10th January 2025. The search strategy was designed to be as comprehensive as possible and was created based on combinations of controlled and uncontrolled search terms. The search strings were adapted to the syntax of each database or platform (Table S1). The reference lists of the studies included were manually checked to identify additional studies. Grey literature was searched using Google Scholar.

### Study selection

EndNote was used to manage the search results and Deduplicator was used for automated deduplication to remove duplicates across databases; the remaining reports were uploaded to Covidence (www.covidence.org/, Melbourne, Victoria, Australia) for screening. Screening of titles and abstracts was conducted by nine of the reviewers divided into four groups. After calibration sessions in which all reviewers independently screened a pilot sample of records (*n* = 3), consistency was controlled and any discrepancies were resolved through discussions within the groups, with the two lead researchers (DJ and DG) arbitrating if needed. Full‐text articles were then obtained for studies that met the inclusion criteria. Rayyan (www.rayyan.ai/, Cambridge, MA, USA) was used for the management of updated searches, with replication of procedures. DeepL Translate (www.deepl.com/en/translator), an artificial intelligence translator, was used to review reports in languages not covered within our team. To minimize translation bias in these cases, an external colleague familiar with the language verified the accuracy of key points. Exclusion reasons were documented and reported at the full‐text stage (Figure S1).

### Data extraction and charting

Detailed requirements for the extraction of data relevant to the study aims were defined in Covidence, including study design and participant details (country, location, diagnostic descriptors, sampling, sample size, ages, sex, and the extent of movement impairments); intervention details (intervention description, interventionists, context, and dosage); and results across outcome measures, along with concerns regarding conflicts of interest or risk of bias. Data extraction was collated in Covidence by members of each group independently. After comparison and agreement, the data included were then exported to Excel. Participants in studies that fulfilled all Diagnostic and Statistical Manual of Mental Disorders, Fifth Edition (DSM‐5) diagnostic criteria were classified as children with DCD, whereas participants in all other studies were categorized as children with sDCD. The types of intervention were divided according to the International Classification of Functioning, Disability and Health (ICF) framework.^13^ Given the substantial heterogeneity across studies in diagnostic practices, intervention types, delivery formats, and outcome measures, we addressed variability through a structured process of data classification and charting. Individual data elements were grouped into broader, conceptually coherent categories based on the expertise and consensus of the multidisciplinary author team. Results were computed according to the content and outcomes of the interventions delivered. Analyses of methodological flaws impeding confidence in the results were categorized as ‘well described’, ‘moderately described’ when not all elements were presented, and ‘unclearly described’ when the content was not presented (Table S2). Excel formulae were used to collate the data.

## RESULTS

### Screening and selection

Database searches yielded 32 420 citations. Additional searches identified 58 studies. After duplicates were removed 19 608 records were screened, resulting in 537 after title and abstract review. Four reports were not retrieved (theses in Chinese) and 326 articles were excluded. Finally, 207 full‐text articles met the inclusion criteria, representing 202 distinct studies, including 206 interventions (Figure S1 and Table S1).

### Sample characteristics

A total of 4593 children with DCD or sDCD participated in the experimental groups. Most studies used the manual of the DSM‐5 (59.4%) to identify children with DCD. Some studies used the Diagnostic and Statistical Manual of Mental Disorders, Fourth Edition (9.4%), the Diagnostic and Statistical Manual of Mental Disorders, Fourth Edition, Text Revision (3.5%), or the International Classification of Diseases, 10th Revision (1.5%). A significant proportion of studies (26.2%) provided insufficient detail regarding the identification of DCD.

Although most studies reported following the DSM‐5 diagnostic criteria, not all four criteria were fulfilled: (1) significant motor skills difficulties (77.2%); (2) interference with everyday activities (45.5%); (3) symptoms present since early age (12.9%); and (4) symptoms not caused by medical or neurological disorders (19.8%). All four criteria were reported in only 10.4% of studies.

For testing criterion 1, the Movement Assessment Battery for Children, Second Edition (56.7%) and the Movement Assessment Battery for Children (12.8%) were used most frequently, followed by the Bruininks–Oseretsky Test of Motor Proficiency, the Test of Gross Motor Development, Second Edition, the McCarron Assessment of Neuromuscular Development, and the Body Coordination Test for Children (Körperkoordinationtest für Kinder) (11.8%). This information was not presented in 23.0%. Six studies reported using more than one tool. For testing criterion 2, the Developmental Coordination Disorder Questionnaire (22.5%), the Developmental Coordination Disorder Questionnaire’07 (7.0%), the Movement Assessment Battery for Children, Second Edition Checklist (2.1%), or non‐specified reports from teachers or parents (7.0%) were used, but were mostly unclear (61.0%). Two studies reported using two tools.

For the remaining analyses, participants in studies who explicitly met all DSM‐5 diagnostic criteria were classified as children with DCD, whereas participants in all other studies were categorized as children with sDCD.

Participants included were divided according to mean age as follows: 3 to 5 years; 6 to 8 years; 9 to 11 years; 12 to 14 years; and 15 to 25 years. Most studies focused on age groups 6 to 8 years (DCD 52.4%; sDCD 49.2%) and 9 to 11 years (DCD 47.6%; sDCD 34.8%), and fewer on age groups 3 to 5 years (only sDCD 8.8%) and 12 to 14 years (only sDCD 7.2%). We found no study within the age range of 15 to 25 years.

Intervention studies were performed in all inhabited continents: Africa (DCD 23.8%; sDCD 7.7%); USA (DCD 33.3%; sDCD 18.2%); Asia (DCD 19.0%; sDCD 41.4%); Australia and Oceania (DCD 14.3%; sDCD 6.1%); and Europe (DCD: 9.5%; sDCD 27.6%). The highest number of studies were conducted in Iran (*n* = 36), followed by Brazil (*n* = 16), Canada (*n* = 16), South Africa (*n* = 16), UK (*n* = 13), Hong Kong (*n* = 11), Australia (*n* = 10), and the Netherlands (*n* = 9), with a further 28 countries with fewer studies. None of the studies included conceptual nor final priorities defined by (the parents of) children with lived experience.

### Methodology

The occurrence of methodological factors that affected clarity of reporting and interpretations were (1) diagnosis: moderately 58.9%, unclear 22.3%; (2) sampling: moderately 16.8%, unclear 8.9%; (3) location: moderately 4.5%, unclear 22.3%; and (4) interventionist: moderately 9.9%, unclear 23.8% (Table S2).

### Interventions

The interventions were defined into 13 categories, spanning from task‐focused to health promotion. The number of interventions reported in each category (some studies included more than one intervention) and the number of participating children are presented in Table 1. Active video games and sensory‐perceptual motor skill training were the most common.

**Table 1:** Intervention categories, number of studies, and participants.

Intervention category	Number of studies		Number of participants in the studies		Total number of participants		Total dose (hours)		Intensity (hours per week)	
	DCD	sDCD	DCD	sDCD	DCD	sDCD	DCD	sDCD	DCD	sDCD
A. Neuromotor task training	1	4	18	5–44	18	96	16	12 (5–27)	1.8	1.8 (1.4–2.3)
B. Cognitive orientation to occupational performance	6	17	9–53	1–78	146	291	10 (7–28)	10 (5–12)	1.9 (1.0–6.7)	1.0 (0.7–9.5)
C. Motor imagery	2	5	8–12	10–40	20	100	9 (5–13)	7 (7–11)	1.2 (1.0–1.4)	1.7 (0.8–2.7)
D. Task‐oriented approach	2	11	20–37	7–47	57	262	10 (6–13)	11 (5–36)	0.8 (0.5–1.0)	1.0 (0.5–3.0)
E. Sensory: perceptual (sensory, motor, visual, auditory) motor skill training	0	30	–	4–199	–	846	–	12 (3–274)	–	1.5 (0.3–6.0)
F. Motor skill training	2	25	13–27	4–98	40	661	31 (25–36)	24 (5–36)	3.3 (3.0–3.6)	2.2 (0.7–3.0)
G. Physical exercise (endurance, strength, and core training)	2	24	30–44	1–58	74	519	35 (16–54)	18 (2–40)	3.1 (1.6–4.5)	2.3 (0.3–3.3)
H. Balance training	0	5	–	2–30	–	83	–	18 (9–28)	–	2.3 (1.7–25.0)
I. Active videogames	4	37	16–35	1–57	85	734	3 (3–10)	9 (1–36)	0.6 (0.6–1.0)	1.4 (0.1–2.5)
J. Sports training (martial arts, soccer, trampoline, horse riding, dance training)	2	16	30	10–60	60	367	54	30 (3–96)	4.5	3.8 (0.5–8.0)
K. Occupational performance coaching, family‐oriented and school‐oriented approach	0	5	–	1–31	–	38	–	7 (4–58)	–	3.8 (1.8–5.8)
L. Handwriting/graphomotor	0	2	–	22–27	–	49	–	7 (4–9)	–	0.5 (0.5–0.6)
M. Fascia Bowen therapy, health promotion, others	2	4	14–22	1–10	36	11	2	5	1.7	0.8

Abbreviations: DCD, developmental coordination disorder; sDCD, suspected developmental coordination disorder.

The delivery formats of individual versus group sessions showed some difference in frequencies: large‐group or class‐based (≥ 10; adult:child ratio = 1:10) in 24.8% (DCD 17.4%; sDCD 25.7%); small‐group‐based (< 10 children; adult:child ratio = 1:3 up to three adults) in 32.0% (DCD 39.1%; sDCD 31.1%); individual sessions (adult:child ratio = 1:1) in 33.0% (DCD 39.1%; sDCD 32.2%) of interventions, but were unclear or not detailed in 10.2% of interventions.

Most interventions (DCD 95.7%; sDCD 86.3%) included supervised sessions with dosage described in hours. Six interventions included only home unsupervised sessions (only sDCD 3.3%); in 20 interventions (DCD 4.3%; sDCD 10.4%), no clear description of dosage was reported. The most used dose was between 1 hour and 10 hours (DCD 40.9% of 22 supervised interventions; sDCD 35.4% of 158 supervised interventions) and between 10 hours and 20 hours (DCD 50.0%; sDCD 34.2%); higher doses were between 20 hours and 30 hours (DCD 4.5%; sDCD 18.4%) or over 30 hours (DCD 4.5%; sDCD 11.4%).

### Outcomes

All outcomes were classified into 11 general outcome measure domains across ICF domains. Table 2 outlines the number of studies across domains and the corresponding categories of outcome measures and ICF domains.

**Table 2:** Frequency of studies observing specific and general outcome measures and ICF domains.

General outcome measures	General outcome measures: frequency of studies		Specific outcome measures	Specific outcome measures: frequency of studies		ICF domain
	DCD	sDCD		DCD	sDCD	
Health mental	3	20	Anxiety	0	3	Body function
			Depression	0	4	
			Enjoyment	2	10	
			Self‐esteem	1	3	
			Self‐perception	0	2	
Health physical	1	15	Fitness	1	6	Body function
			Physiological functions	0	8	
			Body composition	1	3	Body structure
Mental functions	4	27	Brain structure	1	0	Body structure
			Attention	1	6	Body function
			Cognitive functions	1	9	
			Encouragement	0	1	
			Executive functions	1	9	
			Intelligence	0	1	
			Communication	0	1	Activities and participation
			Educational achievement	0	1	
			Reading	0	2	
Sensory functions	1	26	Sensory functions	1	9	Body function
			Sensory functions, hearing	0	3	
			Sensory functions, motor	0	2	
			Sensory functions, tactile	0	5	
			Sensory functions, vestibular	0	1	
			Sensory functions, visual	0	18	
Quality of movement	4	14	Movement performance	1	1	Body function
			Sports performance quality	0	1	
			Task performance quality	3	12	
Motor performance	20	159	Balance	12	76	Body function
			Coordination motor	3	17	
			Fine motor skills	7	70	
			Flexibility	1	1	
			Gross motor skills	6	69	
			Motor performance, general	12	75	
			Motor skills	0	3	
			Speed and agility	4	15	
			Strength	8	25	
			Endurance	1	4	
			Perceived competence	2	8	Activities and participation
			Task performance	5	32	
			Task satisfaction	2	18	
Physical activity	1	8	Physical activity	1	8	Activities and participation
Participation	3	8	Engagement	0	2	Activities and participation
			Participation	1	1	
			Predilection for physical activity	1	3	
			Preferences for activities	0	1	
			Self‐efficacy	1	5	Body function
Environment	3	0	Falls	3	0	Body function
Social skills and relations	1	11	Social responsiveness	0	2	Body function
			Behaviour	0	6	Activities and participation
			Social acceptance	1	1	
			Social relations	0	1	
			Social skills	0	4	
Quality of life	0	3	Quality of life	0	3	Activities and participation

Abbreviations: DCD, developmental coordination disorder; ICF, International Classification of Functioning, Disability and Health; sDCD, suspected developmental coordination disorder.

Outcomes were mapped to the ICF^13^ domains of body structure, body function, and activities and participation. Four studies (*n* = 1 DCD; *n* = 3 sDCD) considered outcomes related to body structure such as nervous system and structures related to movement. Almost all interventions were related to body function (DCD 100%; sDCD 93.9%) and mainly focused on neuromusculoskeletal and movement‐related functions (DCD 95.2%; sDCD 83.4%), and less frequent to mental functions (DCD 22.3%; sDCD 24.3%), functions of the cardiovascular, haematological, immunological, and respiratory systems (DCD 9.5%; sDCD 7.2%), and sensory functions and pain (DCD 4.8%; sDCD 14.4%).

The activities and participation domain (including mobility, communication, general tasks and demands, interpersonal interactions and relationships, learning and applying knowledge) were included in 33.3% of DCD and 34.8% of sDCD studies. Most studies aimed at general task and demands (DCD 33.3%; sDCD 22.1%), and some at interpersonal interactions and relationships (DCD 4.8%; sDCD 6.1%) and mobility (DCD 4.8%; sDCD 5.0%). Learning and applying knowledge (only sDCD 1.7%) and communication (only sDCD 0.6%) were only considered in a few studies (Table 2).

### Effects of interventions

**Figure 1:**
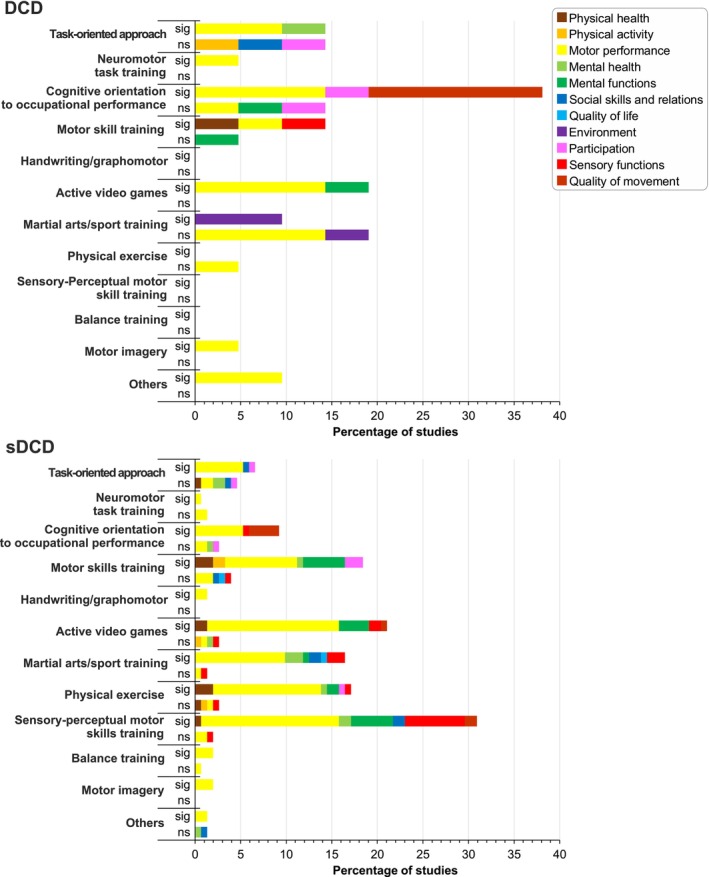
Effects of specific interventions on observed general outcome measures. Abbreviations: DCD, developmental coordination disorder; sDCD, suspected developmental coordination disorder; sig, significant effects reported *p* < 0.05; ns, non significant effects reported.

The studies in our sample differed considerably in statistical processing and effect size evaluations. Therefore, we adopted a relatively simple procedure to indicate the effect of the intervention based on a significant difference between pre‐test and post‐test within the experimental group, or if a significant group × time effect in the analysis of variance was reported. However, this procedure has limitations because it ignores effect sizes and the quality of the study design. Most interventions (DCD 61.9%; sDCD 77.0%) reported a significant improvement of the outcome measure motor performance; in 20 studies (DCD *n* = 5, 23.8%; sDCD *n* = 15, 9.9%), the beneficial change after the intervention was not presented. Results for all interventions and outcome measure domains are presented in Figure 1.

Table 3 presents the general outcome measures per group size delivered and the ratio of studies that presented significant results, which indicates that large and small groups have almost equal ratios between significant and non‐significant results (85% and 82%) compared to individual interventions (81%). Studies that integrated parents showed significant improvement in 60%. However, in all delivery formats, this ratio was smaller for studies with children with DCD compared to studies of children with sDCD.

**Table 3:** Effects of delivery format on observed general outcome measures.

General outcome measures	Large group						Small group						One‐to‐one child only						One‐to‐one with parent					
	DCD		sDCD		Total		DCD		sDCD		Total		DCD		sDCD		Total		DCD		sDCD		Total	
	sig	NS	sig	NS	sig	NS	sig	NS	sig	NS	sig	NS	sig	NS	sig	NS	sig	NS	sig	NS	sig	NS	sig	ns
Environment	2	1	0	0	2	1	0	0	0	0	0	0	0	0	0	0	0	0	0	0	0	0	0	0
Health, mental	0	0	7	0	7	0	0	0	1	3	1	3	1	0	0	1	1	1	0	0	0	0	0	0
Health, physical	0	0	2	0	2	0	1	0	4	1	5	1	0	0	3	1	3	1	0	0	0	0	0	0
Mental functions	0	0	12	0	12	0	0	1	5	0	5	1	1	0	3	0	4	0	0	1	0	0	0	1
Motor performance	1	3	27	5	28	8	7	1	42	4	49	5	4	1	33	5	37	6	1	0	2	1	3	1
Participation	0	0	2	0	2	0	1	0	2	2	3	2	0	1	0	0	0	1	0	1	0	0	0	1
Physical activity	0	0	2	1	2	1	0	0	0	1	0	1	0	1	0	0	0	1	0	0	0	0	0	0
Quality of life	0	0	1	1	1	1	0	0	0	0	0	0	0	0	0	0	0	0	0	0	0	0	0	0
Quality of movement	0	0	2	0	2	0	2	0	1	0	3	0	1	0	3	0	4	0	1	0	1	0	2	0
Sensory functions	0	0	1	0	1	0	1	0	9	2	10	2	0	0	5	1	5	1	0	0	1	1	1	1
Social skills and relations	0	0	2	0	2	0	0	1	1	1	1	2	0	0	1	2	1	2	0	0	0	0	0	0
Total	3	4	58	7	61	11	12	3	65	14	77	17	7	3	48	10	55	13	2	2	4	2	6	4
Ratio (percentage of studies with significant findings)	43		89		85		80		82		82		70		83		81		50		67		60	
95% confidence interval	16	75	79	95	75	91	55	93	72	89	73	88	40	89	71	90	70	88	15	85	30	90	31	83

Sig indicates significant improvement, while NS indicates no significant improvement. Abbreviations: DCD, developmental coordination disorder; sDCD, suspected developmental coordination disorder.

## DISCUSSION

Understanding the complexity of DCD as a condition and its broader impacts has significantly advanced over the past decade, yet evaluation of different types and delivery methods of effective interventions across several contexts remains limited. This study aimed to identify and synthesize the characteristics of interventions provided to children and adolescents with DCD and further explored several administration methods and outcomes. A broad search across health and educational databases identified 202 distinct studies, within which 485 unique outcome measures were reported, reflecting diverse outcome perspectives in different domains.

Our findings show inconsistency among authors in describing how children with coordination difficulties were defined and categorized, including whether full diagnostic criteria for DCD were applied. Although most studies provided clear descriptions of their sampling methods, many offered poor descriptions of the measures used to diagnose DCD, with criterion 1 of the DSM‐5 most commonly met. It is concerning that all four criteria for DCD were only addressed in 10% of studies. We recommend incorporating parental questionnaires in diagnostic formulations because parents' concerns about their children's development and behaviour may offer a more comprehensive view of the child over time. This approach could improve detection rates in paediatric practices^14^, ^15^ and can be easily included by using reliable and valid instruments, with validated cultural translations.^16^, ^17^ By carefully screening all four DSM‐5 criteria for DCD, global comparison of interventions can be undertaken.

A diverse range of interventions was identified, involving 4593 children with DCD or sDCD across multiple countries. This represents a substantial sample size; however, variations in diagnostic criteria and sampling methods between studies limit the comparability of outcomes. Greater alignment in study design could improve the potential to draw robust conclusions about intervention effectiveness across the motor, cognitive, and participation domains. Most interventions primarily targeted children's movement difficulties through task‐oriented training, motor skill practice, or sports‐based physical activity, reflecting an activity‐oriented and participation‐oriented approach that is consistent with current clinical practise recommendations. Some interventions additionally focused on improving physical fitness components, such as cardiovascular endurance, reflecting broader concerns about the physical health of this population.^18^ Others incorporated cognitive strategies and coaching approaches to support improvements in activity performance and participation. However, despite this activity‐focused intervention content, outcome evaluation across studies remains predominantly centred on body functions, with comparatively fewer studies assessing participation‐level outcomes, psychosocial factors, or transfer of gains to real‐life contexts. These findings highlight opportunities to adopt more holistic intervention and evaluation models that integrate cognitive, emotional, and environmental considerations alongside motor outcomes, thereby better supporting meaningful participation across diverse everyday s.

The more recent increase in studies considering quality of life and social–emotional outcomes with the growing recognition of the psychosocial impact of DCD is encouraging.^19^ A recent meta‐analysis reported that children with DCD showed significantly lower overall quality of life than typically developing children; this was found across multiple domains (i.e. physical, emotional, social, and school), highlighting the extent of the impact of DCD.^20^ This is consistent with the shift in perspectives in rehabilitation adopted by the ‘F words’,^8^ to focus not just on ‘function’ and ‘fitness’, but also on ‘friends’, ‘family’, ‘fun’, and ‘future’. Children with greater perceptions of motor competence are reportedly more physically active than children with lower perceived competence.^21^ Additionally, relationships between perceived motor skills and actual motor performance are regarded as primary influencers of children's participation in physical activity.^22^ We recommend that future interventions for children with DCD expand their therapeutic focus to incorporate psychosocial factors, enabling better support for the children's overall development, including areas such as social relationships, family dynamics, and future prospects.

Of interest and note in our findings are the variety of settings in which interventions were delivered. The delivery method of interventions in a large group, small group, or in an individual session all reflected significant benefits on several outcome measures. Most interventions that included parent coaching for their children were delivered in small or large groups within the children's school environment, helping to raise awareness of DCD among both parents and teachers.^23^ Besides advantages of being in typical and familiar environments and training with peers, these types of interventions are more time‐effective and cost‐effective. With the broader educational and community involvement in the studies included, these findings represent encouraging opportunities for access to interventions that may meet needs in differing socioeconomic and culturally diverse populations.^24^, ^25^

The total dosage of intervention sessions varied, with most studies reporting between 1 hour and 10 hours, and 10 hours and 20 hours. Dosage was poorly reported in most studies, yet such information is vital for therapists, physical education teachers, and trainers. We suggest that future research should prioritize providing clear and detailed reporting of intervention dosages to enhance the transferability of findings and support the implementation of evidence‐based practices.

Most interventions (*n* = 130) showed a significant improvement in motor performance, specifically targeting motor coordination, a key issue in DCD. Large‐group interventions presented more environmental and mental health changes, while small‐group and individual interventions presented more changes in physical health, mental functions, and motor performance. The increasing focus on studies addressing environmental, mental health, and cognitive factors is promising because impaired motor functioning in children with DCD also affects cognitive, social, and psychological development, with these challenges continuing into adolescence.^26^, ^27^, ^28^ Importantly, the lack of interventions targeting children older than 15 years is concerning, particularly given that participation outcomes, such as engagement in community clubs, have a prominent role in this age group.

### Strengths and limitations

Our study was limited in its ability to compare differing dosages and duration of therapies and consider their cost‐effectiveness, and hence accessibility and affordability in different socioeconomic settings and cultures, because of lack of information. The high number of studies which included regular sporting, ‘martial arts’, or active video games in the context of children's everyday lives was encouraging.^24^, ^25^, ^29^ This may open access to participate in regular community activities for young people with DCD with some specialist support. Advantages of the wider scope of this review were the opportunities to explore the range of interventions delivered in different contexts and age groups. These identified a large number of interventions that have been offered to children with DCD. Although this approach has its limitations, by including as many studies as possible and only using and evaluating the presented significant changes before and after the intervention, as calculated by the authors of the studies included, we provided recommendations for future interventions, rather than precisely calculating the effects from a small sample of studies.

### Recommendations

Future intervention studies should ensure alignment with all diagnostic criteria to enhance clarity and consistency when including individuals with DCD. Researchers should provide detailed descriptions of interventions, including who delivered them (and qualifications), the type, dose, and duration of training, and the mode and context of delivery. Intervention content should prioritize practising motor skills through a combination of practical and cognitive approaches, emphasizing the transfer of these skills to functional participation in daily life. It should also make sure that not only motor performance, but also participation, psychosocial attention related to quality of life, and perceived competence are addressed. To further strengthen intervention design, additional components of motor learning, including opportunities for autonomy, experiences of relatedness, and strategies that enhance expectancies may be incorporated to promote greater enjoyment, self‐determination, and ultimately intrinsic motivation. In future intervention studies, researchers could involve the experiences of people with lived experience to select outcome measures that are conditional for participation and quality of life. This will support the translation of gains in motor performance to participation and consider that psychosocial and quality of life outcomes are addressed. Moreover, outcome measures should expand to include three critical domains: (1) the practical application of learned motor skills; (2) environmental and mental health impacts; and (3) parental awareness and support. Taken together, to improve transparency and replicability of intervention research in children with DCD, future studies should use standardized reporting frameworks such as the Template for Intervention Description and Replication^30^ or the Consensus on Exercise Reporting Template^31^ checklists. These tools provide structured guidance for documenting intervention components, delivery, and fidelity, thereby facilitating clearer interpretation and comparison across studies.

## Conclusions

This review included 206 interventions, including specialized therapeutic protocols and community‐based activities, which often showed improvements in motor performance. Long‐term psychosocial outcomes and sustained participation benefits remain less explored. To address these gaps, interventions for children with DCD should integrate physical activities with cognitive elements, while considering emotional and environmental factors, to maximize their impact. Involvement of children and young people with lived experience (DCD) and their parents in setting the aims of interventions together and to evaluate processing and experiencing is needed. By adopting comprehensive outcome measures and designing interventions to children's specific needs, future research can ensure that programmes are both time‐effective and affordable, fostering meaningful improvements in both motor abilities and overall well‐being.

## CONFLICT OF INTEREST

No potential conflict of interest was reported by the authors.

## Supporting information


**Figure S1:** PRISMA flowchart.

**Table S1:** Database search strings.

**Table S2:** Evaluation of methodological flaws relative occurrence.

## Data Availability

The data that supports the findings of this study are available in the supplementary material of this article.

## References

[1] Lingam R , Hunt L , Golding J , Jongmans M , Emond A . Prevalence of developmental coordination disorder using the DSM‐IV at 7 years of age: A UK population‐based study. Pediatrics. 2009;123(4):e693–e700. 10.1542/peds.2008-1770. 19336359

[2] American Psychiatric Association . Diagnostic and statistical manual of mental disorders, fifth edition. Washington, DC: American Psychiatric Publishing, Inc., 2013. 10.1176/appi.books.9780890425596.

[3] Blank R , Barnett AL , Cairney J , Green D , Kirby A , Polatajko H , et al. International clinical practice recommendations on the definition, diagnosis, assessment, intervention, and psychosocial aspects of developmental coordination disorder. Dev Med Child Neurol. 2019;61(3):242–85. 10.1111/dmcn.14132. 30671947 PMC6850610

[4] Dannemiller L , Mueller M , Leitner A , Iverson E , Kaplan SL . Physical Therapy Management of Children with Developmental Coordination Disorder: An Evidence‐Based Clinical Practice Guideline from the Academy of Pediatric Physical Therapy of the American Physical Therapy Association. Pediatr Phys Ther. 2020;32(4):278–313. 10.1097/PEP.0000000000000753. 32991554

[5] Kirby A , Williams N , Thomas M , Hill EL . Self‐reported mood, general health, wellbeing and employment status in adults with suspected DCD. Res Dev Disabil. 2013;34(4):1357–64. 10.1016/j.ridd.2013.01.003. 23417140

[6] Smits‐Engelsman B , Vinçon S , Blank R , Quadrado VH , Polatajko H , Wilson PH . Evaluating the evidence for motor‐based interventions in developmental coordination disorder: A systematic review and meta‐analysis. Res Dev Disabil. 2018;74:72–102. 10.1016/j.ridd.2018.01.002. 29413431

[7] D'Hondt E , Venetsanou F , Kambas A , Lenoir M . Motor competence levels in young children: A cross‐cultural comparison between Belgium and Greece. J Mot Learn Dev. 2019;7(3):289–306. 10.1123/jmld.2018-0044.

[8] Rosenbaum P , Gorter JW . The ‘F‐words’ in childhood disability: I swear this is how we should think. Child Care Health Dev. 2012;38(4):457–63. 10.1111/j.1365-2214.2011.01338.x. 22040377

[9] Grant MJ , Booth A . A typology of reviews: An analysis of 14 review types and associated methodologies. Health Info Libr J. 2009;26(2):91–108. 10.1111/j.1471-1842.2009.00848.x. 19490148

[10] Arksey H , O'Malley L . Scoping studies: Towards a methodological framework. Int J Soc Res. 2005;8(1):19–32. 10.1080/1364557032000119616.

[11] Peters MDJ , Marnie C , Tricco AC , Pollock D , Munn Z , Alexander L , et al. Updated methodological guidance for the conduct of scoping reviews. JBI Evid, Synth. 2020;18(10):2119–26. 10.11124/JBIES-20-00167. 33038124

[12] Tricco AC , Lillie E , Zarin W , O'Brien KK , Colquhoun H , Levac D , et al. PRISMA extension for scoping reviews (PRISMA‐ScR): Checklist and explanation. Ann Intern Med. 2018;169(7):467–73. 10.7326/M18-0850. 30178033

[13] World Health Organization . International Classification of Functioning, Disability, and Health: Children & Youth Version: ICF‐CY: World Health Organization, 2007,2024.

[14] Glascoe FP . The value of parents' concerns to detect and address developmental and behavioural problems. J Paediatr Child Health. 1999;35(1):1–8. 10.1046/j.1440-1754.1999.00342.x.

[15] Green D , Bishop T , Wilson BN , Crawford S , Hooper R , Kaplan B , et al. Is questionnaire‐based screening part of the solution to waiting lists for children with developmental coordination disorder? Br J Occup Ther. 2005;68(1):2–10. 10.1177/030802260506800102.

[16] Schoemaker MM , Flapper BCT , Reinders‐Messelink HA , Kloet Ad . Validity of the motor observation questionnaire for teachers as a screening instrument for children at risk for developmental coordination disorder. Hum Mov Sci. 2008;27(2):190–9. 10.1016/j.humov.2008.02.003. 18346804

[17] Wilson BN , Crawford SG , Green D , Roberts G , Aylott A , Kaplan BJ . Psychometric properties of the revised developmental coordination disorder questionnaire. Phys Occup Ther Pediatr. 2009;29(2):182–202. 10.1080/01942630902784761. 19401931

[18] Caçola P. Physical and mental health of children with developmental coordination disorder. Front Publ Health. 2016;4(OCT). 10.3389/FPUBH.2016.00224. PMC5075567 27822464

[19] O'Dea Á , Connell A . Performance difficulties, activity limitations and participation restrictions of adolescents with developmental coordination disorder (DCD). Br J Occup Ther. 2016;79(9):540–9. 10.1177/0308022616643100.

[20] Kumar G , Barhoun P , Fuelscher I , Zwicker JG , Bianco K , Singh M , et al. Quality of life in children with developmental coordination disorder (DCD): A systematic review and meta‐analysis. Res Dev Disabil. 2025;160. 10.1016/j.ridd.2025.104981. 40121827

[21] Mancini VO , Rigoli D , Roberts LD , Heritage B , Piek JP . The relationship between motor skills and psychosocial factors in young children: A test of the elaborated environmental stress hypothesis. Br J Educ Psychol. 2018;88(3):363–79. 10.1111/bjep.12187. 28884809

[22] Zhang T , Thomas K , Weiller K . Predicting physical activity in 10–12 year old children: A social ecological approach. J Teach Phys Educ. 2015;34(3):517–36. 10.1123/jtpe.2013-0195.

[23] Steenbergen B , Valtr L , Dunford C , Prunty M , Bekhuis H , Temlali TY , et al. Awareness about developmental coordination disorder. Front Publ Health. 2024;12. 10.3389/fpubh.2024.1345257. PMC10867205 38362216

[24] Fong SSM , Chung LMY , Schooling CM , Lau EHY , Wong JYH , Bae YH , et al. Tai chi‐muscle power training for children with developmental coordination disorder: A randomized controlled trial. Sci Rep. 2022;12(1). 10.1038/s41598-022-25822-x. PMC9769475 36543796

[25] Ghadiri F , O'Brien W , Soltani S , Faraji M , Bahmani M . The effect of karate interventions on the motor proficiency of female adolescents with developmental coordination disorder (DCD) from high and low socio‐economic status. BMC Sports Sci Med Rehabil. 2022;14(1). 10.1186/s13102-022-00501-6. PMC9258153 35794627

[26] Green D , Baird G , Sugden D . A pilot study of psychopathology in Developmental Coordination Disorder. Child Care Health Dev. 2006;32(6):741–50. 10.1111/j.1365-2214.2006.00684.x. 17018049

[27] Harrowell I , Hollén L , Lingam R , Emond A . Mental health outcomes of developmental coordination disorder in late adolescence. Dev Med Child Neurol. 2017;59(9):973–9. 10.1111/dmcn.13469. 28512766 PMC5573907

[28] Missiuna C , Moll S , King G , Stewart D , Macdonald K . Life experiences of young adults who have coordination difficulties. Can J Occup Ther. 2008;75(3):157–66. 10.1177/000841740807500307. 18615927

[29] Jelsma LD , Cavalcante Neto JL , Smits‐Engelsman B , Targino Gomes Draghi T , Araújo Rohr L , Tudella E . Type of active video‐games training does not impact the effect on balance and agility in children with and without developmental coordination disorder: A randomized comparator‐controlled trial. Appl Neuropsychol Child. 2023;12(1):64–73. 10.1080/21622965.2022.2030740. 35098823

[30] Hoffmann TC , Glasziou PP , Boutron I , Milne R , Perera R , Moher D , et al. Better reporting of interventions: Template for intervention description and replication (TIDieR) checklist and guide. BMJ. 2014;348. 10.1136/bmj.g1687. 24609605

[31] Slade SC , Dionne CE , Underwood M , Buchbinder R , Beck B , Bennell K , et al. Consensus on exercise reporting template (CERT): Modified Delphi study. Phys Ther. 2016;96(10):1514–24. 10.2522/ptj.20150668. 27149962

