# Supplementary material for: Interventions for children with developmental coordination disorder: A scoping review
Source: Dev Med Child Neurol. 2026 Feb 26;68(9):1184–94. doi: 10.1111/dmcn.70221 (PMC13436707; doi:10.1111/dmcn.70221)
Supplement: Supplementary file 1 [file DMCN-68-1184-s001.docx]

**Table S2** Evaluation of methodological flaws relative occurrence (in %)

| Methodological flaws | Unclearly described | Moderately described | Well described |
| --- | --- | --- | --- |
| Diagnosis | 22.3 | 58.9 | 18.8 |
| Sampling | 8.9 | 16.8 | 74.3 |
| Intervention location | 22.3 | 4.5 | 73.3 |
| Trainer/therapist | 23.8 | 9.9 | 66.3 |
