# Supplementary material for: Interventions for children with developmental coordination disorder: A scoping review
Source: Dev Med Child Neurol. 2026 Feb 26;68(9):1184–94. doi: 10.1111/dmcn.70221 (PMC13436707; doi:10.1111/dmcn.70221)
Supplement: Supplementary file 2 [file DMCN-68-1184-s002.docx]

# Evidence-based practice interventions for children and young people with Developmental Coordination Disorder - A scoping review

**Table S1. Database search strings**

AMED (EBSCOhost) n=**522**
Date of search: January 10, 2025
Search mode: Advanced Search/Proximity

|  | **Query** | **Results** |
| --- | --- | --- |
| S1 | TI apraxia OR ”atypical brain development” OR ”clumsy child*” OR DAMP OR dcd OR ”deficits in attention motor control and perception” OR ”developmental coordination disorder*” OR ”developmental motor coordination disorder” OR dyscoordination OR dyspraxia OR in-coordination OR ”lower motor competence*” OR ”mild motor problem*” OR ”minimal brain damage*” OR ”minimal brain dysfunction” OR ”minimal cerebral dysfunction” OR ”minimal neurological dysfunction” OR ”minor neurological dysfunction” OR ”motor coordination difficult*” OR ”motor coordination problem*” OR ”motor delay disorder*” OR ”motor learning difficult*” OR ”motor skills disorder*” OR ”movement difficult*” OR ”neurodevelopmental disorder*” OR ”non-verbal learning disorder*” OR ”perceptual motor dysfunction*” OR ”perceptual motor impairment” OR ”physical* awkward*” OR ”psychomotor disorder*” OR ”sensory integrative disorder*” OR ”sensory motor difficult*” OR SPDDMF OR ”specific developmental disorder of motor function*” | 1,068 |
| S2 | AB apraxia OR ”atypical brain development” OR ”clumsy child*” OR DAMP OR dcd OR ”deficits in attention motor control and perception” OR ”developmental coordination disorder*” OR ”developmental motor coordination disorder” OR dyscoordination OR dyspraxia OR in-coordination OR ”lower motor competence*” OR ”mild motor problem*” OR ”minimal brain damage*” OR ”minimal brain dysfunction” OR ”minimal cerebral dysfunction” OR ”minimal neurological dysfunction” OR ”minor neurological dysfunction” OR ”motor coordination difficult*” OR ”motor coordination problem*” OR ”motor delay disorder*” OR ”motor learning difficult*” OR ”motor skills disorder*” OR ”movement difficult*” OR ”neurodevelopmental disorder*” OR ”non-verbal learning disorder*” OR ”perceptual motor dysfunction*” OR ”perceptual motor impairment” OR ”physical* awkward*” OR ”psychomotor disorder*” OR ”sensory integrative disorder*” OR ”sensory motor difficult*” OR SPDDMF OR ”specific developmental disorder of motor function*” | 2,132 |
| S3 | SU apraxia OR ”atypical brain development” OR ”clumsy child*” OR DAMP OR dcd OR ”deficits in attention motor control and perception” OR ”developmental coordination disorder*” OR ”developmental motor coordination disorder” OR dyscoordination OR dyspraxia OR in-coordination OR ”lower motor competence*” OR ”mild motor problem*” OR ”minimal brain damage*” OR ”minimal brain dysfunction” OR ”minimal cerebral dysfunction” OR ”minimal neurological dysfunction” OR ”minor neurological dysfunction” OR ”motor coordination difficult*” OR ”motor coordination problem*” OR ”motor delay disorder*” OR ”motor learning difficult*” OR ”motor skills disorder*” OR ”movement difficult*” OR ”neurodevelopmental disorder*” OR ”non-verbal learning disorder*” OR ”perceptual motor dysfunction*” OR ”perceptual motor impairment” OR ”physical* awkward*” OR ”psychomotor disorder*” OR ”sensory integrative disorder*” OR ”sensory motor difficult*” OR SPDDMF OR ”specific developmental disorder of motor function*” | 1,984 |
| S4 | S1 OR S2 OR S3 | 4,182 |
| S5 | TI “active video gam*” OR “attention* focus” OR AVG OR “cognitive orientation to daily occupational performance*” OR CO-OP OR “CO OP” OR danc* OR exercise* OR “handwriting teaching” OR imagery OR intervention* OR karate OR “motivation* learning” OR ”motivation* coaching” OR “motor learning” OR “motor performance” OR OT OR PA OR PE OR “physical activit*” OR “physical education” OR physiotherap* OR play* OR rehabilit* OR sport* OR therap* OR train* OR treatment* OR yoga | 85,841 |
| S6 | AB “active video gam*” OR “attention* focus” OR AVG OR “cognitive orientation to daily occupational performance*” OR CO-OP OR “CO OP” OR danc* OR exercise* OR “handwriting teaching” OR imagery OR intervention* OR karate OR “motivation* learning” OR ”motivation* coaching” OR “motor learning” OR “motor performance” OR OT OR PA OR PE OR “physical activit*” OR “physical education” OR physiotherap* OR play* OR rehabilit* OR sport* OR therap* OR train* OR treatment* OR yoga | 122,532 |
| S7 | SU “active video gam*” OR “attention* focus” OR AVG OR “cognitive orientation to daily occupational performance*” OR CO-OP OR “CO OP” OR danc* OR exercise* OR “handwriting teaching” OR imagery OR intervention* OR karate OR “motivation* learning” OR ”motivation* coaching” OR “motor learning” OR “motor performance” OR OT OR PA OR PE OR “physical activit*” OR “physical education” OR physiotherap* OR play* OR rehabilit* OR sport* OR therap* OR train* OR treatment* OR yoga | 206,782 |
| S8 | S5 OR S6 OR S7 | 245,856 |
| S9 | TI adolescen* OR child* OR kid* OR school-age* OR ”school age*” OR teenag* OR “young adult*” OR youth | 18,511 |
| S10 | AB adolescen* OR child* OR kid* OR school-age* OR ”school age*” OR teenag* OR “young adult*” OR youth | 19,506 |
| S11 | SU adolescen* OR child* OR kid* OR school-age* OR ”school age*” OR teenag* OR “young adult*” OR youth | 27,924 |
| S12 | S9 OR S10 OR S11 | 36,295 |
| S13 | S4 AND S8 AND S12 | 1,051 |
| S5 | Limit to: Published Date: 2012- | 522 |

APA PsycInfo (ProQuest) n=4,890
Date of search: January 10, 2025
Search mode: Advanced search

|  | **Query** | **Results** |
| --- | --- | --- |
| S1 | tiab(apraxia OR ”atypical brain development” OR ”clumsy child*” OR DAMP OR dcd OR ”deficits in attention motor control and perception” OR ”developmental coordination disorder*” OR ”developmental motor coordination disorder” OR dyscoordination OR dyspraxia OR in-coordination OR ”lower motor competence*” OR ”mild motor problem*” OR ”minimal brain damage*” OR ”minimal brain dysfunction” OR ”minimal cerebral dysfunction” OR ”minimal neurological dysfunction” OR ”minor neurological dysfunction” OR ”motor coordination difficult*” OR ”motor coordination problem*” OR ”motor delay disorder*” OR ”motor learning difficult*” OR ”motor skills disorder*” OR ”movement difficult*” OR ”neurodevelopmental disorder*” OR ”non-verbal learning disorder*” OR ”perceptual motor dysfunction*” OR ”perceptual motor impairment” OR ”physical* awkward*” OR ”psychomotor disorder*” OR ”sensory integrative disorder*” OR ”sensory motor difficult*” OR SPDDMF OR ”specific developmental disorder of motor function*”) | 14,476 |
| S2 | MJMAINSUBJECT.EXACT("Dyspraxia") OR MJMAINSUBJECT.EXACT("Motor Skills") OR MJMAINSUBJECT.EXACT("Neurodevelopmental Disorders") OR MJMAINSUBJECT.EXACT("Psychomotor Development") | 17,991 |
| S3 | S1 OR S2 | 29,166 |
| S4 | tiab(“active video gam*” OR “attention* focus” OR AVG OR “cognitive orientation to daily occupational performance*” OR CO-OP OR “CO OP” OR danc* OR exercise* OR “handwriting teaching” OR imagery OR intervention* OR karate OR “motivation* learning” OR ”motivation* coaching” OR “motor learning” OR “motor performance” OR OT OR PA OR PE OR “physical activit*” OR “physical education” OR physiotherap* OR play* OR rehabilit* OR sport* OR therap* OR train* OR treatment* OR yoga) | 1,828,836 |
| S5 | MJMAINSUBJECT.EXACT("Attention") OR MJMAINSUBJECT.EXACT("Dance") OR MJMAINSUBJECT.EXACT("Martial Arts") OR MJMAINSUBJECT.EXACT("Motivation") OR MJMAINSUBJECT.EXACT("Occupational Therapy") OR MJMAINSUBJECT.EXACT("Physical Activity") OR MJMAINSUBJECT.EXACT("Physical Education") OR MJMAINSUBJECT.EXACT("Physical Therapy") OR MJMAINSUBJECT.EXACT("Rehabilitation") OR MJMAINSUBJECT.EXACT("Sports") OR MJMAINSUBJECT.EXACT("Treatment") OR MJMAINSUBJECT.EXACT("Yoga") | 288,112 |
| S6 | S4 OR S5 | 1,951,128 |
| S7 | S3 AND S6 | 13,727 |
| S8 | Limit to: Publication date: 2012-  Age group: Childhood (birth-12 yrs) OR Adulthood (18 yrs & older) OR School Age (6-12 yrs) OR Adolescence (13-17 yrs) OR Preschool Age (2-5 yrs) OR Young Adulthood (18-29 yrs | 4,890 |

## CINAHL with Full Text (EBSCOhost) n=2,012 Date of search: January 10, 2025 Search mode: Advanced Search/Proximity

|  | **Query** | **Results** |
| --- | --- | --- |
| S1 | TI apraxia OR ”atypical brain development” OR ”clumsy child*” OR DAMP OR dcd OR ”deficits in attention motor control and perception” OR ”developmental coordination disorder*” OR ”developmental motor coordination disorder” OR dyscoordination OR dyspraxia OR in-coordination OR ”lower motor competence*” OR ”mild motor problem*” OR ”minimal brain damage*” OR ”minimal brain dysfunction” OR ”minimal cerebral dysfunction” OR ”minimal neurological dysfunction” OR ”minor neurological dysfunction” OR ”motor coordination difficult*” OR ”motor coordination problem*” OR ”motor delay disorder*” OR ”motor learning difficult*” OR ”motor skills disorder*” OR ”movement difficult*” OR ”neurodevelopmental disorder*” OR ”non-verbal learning disorder*” OR ”perceptual motor dysfunction*” OR ”perceptual motor impairment” OR ”physical* awkward*” OR ”psychomotor disorder*” OR ”sensory integrative disorder*” OR ”sensory motor difficult*” OR SPDDMF OR ”specific developmental disorder of motor function*” | 3,243 |
| S2 | AB apraxia OR ”atypical brain development” OR ”clumsy child*” OR DAMP OR dcd OR ”deficits in attention motor control and perception” OR ”developmental coordination disorder*” OR ”developmental motor coordination disorder” OR dyscoordination OR dyspraxia OR in-coordination OR ”lower motor competence*” OR ”mild motor problem*” OR ”minimal brain damage*” OR ”minimal brain dysfunction” OR ”minimal cerebral dysfunction” OR ”minimal neurological dysfunction” OR ”minor neurological dysfunction” OR ”motor coordination difficult*” OR ”motor coordination problem*” OR ”motor delay disorder*” OR ”motor learning difficult*” OR ”motor skills disorder*” OR ”movement difficult*” OR ”neurodevelopmental disorder*” OR ”non-verbal learning disorder*” OR ”perceptual motor dysfunction*” OR ”perceptual motor impairment” OR ”physical* awkward*” OR ”psychomotor disorder*” OR ”sensory integrative disorder*” OR ”sensory motor difficult*” OR SPDDMF OR ”specific developmental disorder of motor function*” | 6,347 |
| S3 | (MM "Motor Skills Disorders") OR (MM "Psychomotor Disorders") | 2,866 |
| S4 | S1 OR S2 OR S3 | 9,562 |
| S5 | TI “active video gam*” OR “attention* focus” OR AVG OR “cognitive orientation to daily occupational performance*” OR CO-OP OR “CO OP” OR danc* OR exercise* OR “handwriting teaching” OR imagery OR intervention* OR karate OR “motivation* learning” OR ”motivation* coaching” OR “motor learning” OR “motor performance” OR OT OR PA OR PE OR “physical activit*” OR “physical education” OR physiotherap* OR play* OR rehabilit* OR sport* OR therap* OR train* OR treatment* OR yoga | 984,128 |
| S6 | AB “active video gam*” OR “attention* focus” OR AVG OR “cognitive orientation to daily occupational performance*” OR CO-OP OR “CO OP” OR danc* OR exercise* OR “handwriting teaching” OR imagery OR intervention* OR karate OR “motivation* learning” OR ”motivation* coaching” OR “motor learning” OR “motor performance” OR OT OR PA OR PE OR “physical activit*” OR “physical education” OR physiotherap* OR play* OR rehabilit* OR sport* OR therap* OR train* OR treatment* OR yoga | 1,948,718 |
| S7 | (MM "Attention") OR (MM "Dancing") OR (MM "Martial Arts") OR (MM "Motivation") OR (MM "Occupational Therapy") OR (MM "Physical Activity") OR (MM "Physical Education and Training") OR (MM "Physical Therapy") OR (MM "Rehabilitation") OR (MM "Sports") OR (MM "Therapeutics") OR (MM "Telerehabilitation") OR (MM "Yoga") | 132,593 |
| S8 | S5 OR S6 OR S7 | 2,459,016 |
| S9 | S4 AND S8 | 4,513 |
| S10 | Limit to: Publication Date: 2012-  Narrow by SubjectAge: - adult: 19-44 years  Narrow by SubjectAge: - child, preschool: 2-5 years  Narrow by SubjectAge: - all adult  Narrow by SubjectAge: - adolescent: 13-18 years  Narrow by SubjectAge: - child: 6-12 years  Narrow by SubjectAge: - all child | 2,012 |

Education Collection (ProQuest) n=843
Including: Education Database, ERIC
Search mode: Advanced search
Date of search: January 10, 2025

|  | **Query** | **Results** |
| --- | --- | --- |
| S1 | title(apraxia OR ”atypical brain development” OR ”clumsy child*” OR DAMP OR dcd OR ”deficits in attention motor control and perception” OR ”developmental coordination disorder*” OR ”developmental motor coordination disorder” OR dyscoordination OR dyspraxia OR in-coordination OR ”lower motor competence*” OR ”mild motor problem*” OR ”minimal brain damage*” OR ”minimal brain dysfunction” OR ”minimal cerebral dysfunction” OR ”minimal neurological dysfunction” OR ”minor neurological dysfunction” OR ”motor coordination difficult*” OR ”motor coordination problem*” OR ”motor delay disorder*” OR ”motor learning difficult*” OR ”motor skills disorder*” OR ”movement difficult*” OR ”neurodevelopmental disorder*” OR ”non-verbal learning disorder*” OR ”perceptual motor dysfunction*” OR ”perceptual motor impairment” OR ”physical* awkward*” OR ”psychomotor disorder*” OR ”sensory integrative disorder*” OR ”sensory motor difficult*” OR SPDDMF OR ”specific developmental disorder of motor function*” ) | 1,263 |
| S2 | abstract(apraxia OR "atypical brain development" OR "clumsy child*" OR DAMP OR dcd OR "deficits in attention motor control and perception" OR "developmental coordination disorder*" OR "developmental motor coordination disorder" OR dyscoordination OR dyspraxia OR in-coordination OR "lower motor competence*" OR "mild motor problem*" OR "minimal brain damage*" OR "minimal brain dysfunction" OR "minimal cerebral dysfunction" OR "minimal neurological dysfunction" OR "minor neurological dysfunction" OR "motor coordination difficult*" OR "motor coordination problem*" OR "motor delay disorder*" OR "motor learning difficult*" OR "motor skills disorder*" OR "movement difficult*" OR "neurodevelopmental disorder*" OR "non-verbal learning disorder*" OR "perceptual motor dysfunction*" OR "perceptual motor impairment" OR "physical* awkward*" OR "psychomotor disorder*" OR "sensory integrative disorder*" OR "sensory motor difficult*" OR SPDDMF OR "specific developmental disorder of motor function*") | 3,030 |
| S3 | subject(apraxia OR "atypical brain development" OR "clumsy child*" OR DAMP OR dcd OR "deficits in attention motor control and perception" OR "developmental coordination disorder*" OR "developmental motor coordination disorder" OR dyscoordination OR dyspraxia OR in-coordination OR "lower motor competence*" OR "mild motor problem*" OR "minimal brain damage*" OR "minimal brain dysfunction" OR "minimal cerebral dysfunction" OR "minimal neurological dysfunction" OR "minor neurological dysfunction" OR "motor coordination difficult*" OR "motor coordination problem*" OR "motor delay disorder*" OR "motor learning difficult*" OR "motor skills disorder*" OR "movement difficult*" OR "neurodevelopmental disorder*" OR "non-verbal learning disorder*" OR "perceptual motor dysfunction*" OR "perceptual motor impairment" OR "physical* awkward*" OR "psychomotor disorder*" OR "sensory integrative disorder*" OR "sensory motor difficult*" OR SPDDMF OR "specific developmental disorder of motor function*") | 2,427 |
| S4 | S1 OR S2 OR S3 | 4,559 |
| S5 | title(“active video gam*” OR “attention* focus” OR AVG OR “cognitive orientation to daily occupational performance*” OR CO-OP OR “CO OP” OR danc* OR exercise* OR “handwriting teaching” OR imagery OR intervention* OR karate OR “motivation* learning” OR ”motivation* coaching” OR “motor learning” OR “motor performance” OR OT OR PA OR PE OR “physical activit*” OR “physical education” OR physiotherap* OR play* OR rehabilit* OR sport* OR therap* OR train* OR treatment* OR yoga) | 392,132 |
| S6 | abstract("active video gam*" OR "attention* focus" OR AVG OR "cognitive orientation to daily occupational performance*" OR CO-OP OR "CO OP" OR danc* OR exercise* OR "handwriting teaching" OR imagery OR intervention* OR karate OR "motivation* learning" OR "motivation* coaching" OR "motor learning" OR "motor performance" OR OT OR PA OR PE OR "physical activit*" OR "physical education" OR physiotherap* OR play* OR rehabilit* OR sport* OR therap* OR train* OR treatment* OR yoga) | 766,787 |
| S7 | subject("active video gam*" OR "attention* focus" OR AVG OR "cognitive orientation to daily occupational performance*" OR CO-OP OR "CO OP" OR danc* OR exercise* OR "handwriting teaching" OR imagery OR intervention* OR karate OR "motivation* learning" OR "motivation* coaching" OR "motor learning" OR "motor performance" OR OT OR PA OR PE OR "physical activit*" OR "physical education" OR physiotherap* OR play* OR rehabilit* OR sport* OR therap* OR train* OR treatment* OR yoga) | 883,087 |
| S8 | S5 OR S6 OR S7 | 1,477,954 |
| S9 | title(adolescen* OR child* OR kid* OR school-age* OR ”school age*” OR teenag* OR “young adult*” OR youth) | 421,553 |
| S10 | abstract(adolescen* OR child* OR kid* OR school-age* OR ”school age*” OR teenag* OR “young adult*” OR youth) | 694,640 |
| S11 | subject(adolescen* OR child* OR kid* OR school-age* OR ”school age*” OR teenag* OR “young adult*” OR youth) | 1,127,939 |
| S12 | S9 OR S10 OR 11 | 1,400,088 |
| S13 | S4 AND S8 AND S12 | 1,533 |
| S14 | Limit to: Publication date: 2012- | 843 |

MEDLINE (EBSCOhost) n=**5,132**
Date of search: January 10, 2025
Search mode: Advanced Search/Proximity

|  | **Query** | **Results** |
| --- | --- | --- |
| S1 | TI apraxia OR ”atypical brain development” OR ”clumsy child*” OR DAMP OR dcd OR ”deficits in attention motor control and perception” OR ”developmental coordination disorder*” OR ”developmental motor coordination disorder” OR dyscoordination OR dyspraxia OR in-coordination OR ”lower motor competence*” OR ”mild motor problem*” OR ”minimal brain damage*” OR ”minimal brain dysfunction” OR ”minimal cerebral dysfunction” OR ”minimal neurological dysfunction” OR ”minor neurological dysfunction” OR ”motor coordination difficult*” OR ”motor coordination problem*” OR ”motor delay disorder*” OR ”motor learning difficult*” OR ”motor skills disorder*” OR ”movement difficult*” OR ”neurodevelopmental disorder*” OR ”non-verbal learning disorder*” OR ”perceptual motor dysfunction*” OR ”perceptual motor impairment” OR ”physical* awkward*” OR ”psychomotor disorder*” OR ”sensory integrative disorder*” OR ”sensory motor difficult*” OR SPDDMF OR ”specific developmental disorder of motor function*” | 8,906 |
| S2 | AB apraxia OR ”atypical brain development” OR ”clumsy child*” OR DAMP OR dcd OR ”deficits in attention motor control and perception” OR ”developmental coordination disorder*” OR ”developmental motor coordination disorder” OR dyscoordination OR dyspraxia OR in-coordination OR ”lower motor competence*” OR ”mild motor problem*” OR ”minimal brain damage*” OR ”minimal brain dysfunction” OR ”minimal cerebral dysfunction” OR ”minimal neurological dysfunction” OR ”minor neurological dysfunction” OR ”motor coordination difficult*” OR ”motor coordination problem*” OR ”motor delay disorder*” OR ”motor learning difficult*” OR ”motor skills disorder*” OR ”movement difficult*” OR ”neurodevelopmental disorder*” OR ”non-verbal learning disorder*” OR ”perceptual motor dysfunction*” OR ”perceptual motor impairment” OR ”physical* awkward*” OR ”psychomotor disorder*” OR ”sensory integrative disorder*” OR ”sensory motor difficult*” OR SPDDMF OR ”specific developmental disorder of motor function*” | 37,572 |
| S3 | (MM "Motor Skills Disorders") OR (MM "Psychomotor Disorders") OR (MM "Neurodevelopmental Disorders") | 9,950 |
| S4 | S1 OR S2 OR S3 | 46,435 |
| S5 | TI “active video gam*” OR “attention* focus” OR AVG OR “cognitive orientation to daily occupational performance*” OR CO-OP OR “CO OP” OR danc* OR exercise* OR “handwriting teaching” OR imagery OR intervention* OR karate OR “motivation* learning” OR ”motivation* coaching” OR “motor learning” OR “motor performance” OR OT OR PA OR PE OR “physical activit*” OR “physical education” OR physiotherap* OR play* OR rehabilit* OR sport* OR therap* OR train* OR treatment* OR yoga | 3,104,692 |
| S6 | AB “active video gam*” OR “attention* focus” OR AVG OR “cognitive orientation to daily occupational performance*” OR CO-OP OR “CO OP” OR danc* OR exercise* OR “handwriting teaching” OR imagery OR intervention* OR karate OR “motivation* learning” OR ”motivation* coaching” OR “motor learning” OR “motor performance” OR OT OR PA OR PE OR “physical activit*” OR “physical education” OR physiotherap* OR play* OR rehabilit* OR sport* OR therap* OR train* OR treatment* OR yoga | 9,877,311 |
| S7 | (MM "Attention") OR (MM "Dancing") OR (MM "Exercise") OR (MM "Martial Arts") OR (MM "Motivation") OR (MM "Occupational Therapy") OR (MM "Physical Education and Training") OR (MM "Physical Therapy Modalities") OR (MM "Rehabilitation") OR (MM "Sports") OR (MM "Therapeutics") OR (MM "Telerehabilitation") OR (MM "Yoga") | 272,066 |
| S8 | S5 OR S6 OR S7 | 11,055,655 |
| S9 | S4 AND S8 | 18,627 |
|  | Limiters - Published Date: 2012-  Narrow by SubjectAge: - child, preschool: 2-5 years  Narrow by SubjectAge: - young adult: 19-24 years  Narrow by SubjectAge: - adult: 19-44 years  Narrow by SubjectAge: - adolescent: 13-18 years  Narrow by SubjectAge: - all adult: 19+ years  Narrow by SubjectAge: - child: 6-12 years  Narrow by SubjectAge: - all child: 0-18 years | 5,132 |

Scopus (Elsevier) n=11,718
Date of search: January 10, 2025
Search mode: Advanced search

|  | **Query** | **Results** |
| --- | --- | --- |
| S1 | TITLE-ABS-KEY ( apraxia OR "atypical brain development" OR "clumsy child*" OR damp OR dcd OR "deficits in attention motor control and perception" OR "developmental coordination disorder*" OR "developmental motor coordination disorder" OR dyscoordination OR dyspraxia OR in-coordination OR "lower motor competence*" OR "mild motor problem*" OR "minimal brain damage*" OR "minimal brain dysfunction" OR "minimal cerebral dysfunction" OR "minimal neurological dysfunction" OR "minor neurological dysfunction" OR "motor coordination difficult*" OR "motor coordination problem*" OR "motor delay disorder*" OR "motor learning difficult*" OR "motor skills disorder*" OR "movement difficult*" OR "neurodevelopmental disorder*" OR "non-verbal learning disorder*" OR "perceptual motor dysfunction*" OR "perceptual motor impairment" OR "physical* awkward*" OR "psychomotor disorder*" OR "sensory integrative disorder*" OR "sensory motor difficult*" OR spddmf OR "specific developmental disorder of motor function*" ) | 95,053 |
| S2 | TITLE-ABS-KEY ( "active video gam*" OR "attention* focus" OR avg OR "cognitive orientation to daily occupational performance*" OR co-op OR "CO OP" OR danc* OR exercise* OR "handwriting teaching" OR imagery OR intervention* OR karate OR "motivation* learning" OR "motivation* coaching" OR "motor learning" OR "motor performance" OR ot OR pa OR pe OR "physical activit*" OR "physical education" OR physiotherap* OR play* OR rehabilit* OR sport* OR therap* OR train* OR treatment* OR yoga ) | 21,852,861 |
| S3 | TITLE-ABS-KEY ( adolescen* OR child* OR kid* OR school-age* OR "school age*" OR teenag* OR "young adult*" OR youth ) | 7,488,824 |
| S4 | S1 AND S2 AND S3 | 16,217 |
| S5 | Limit to: PubYear: 2012- | 11,718 |

**Web of Science Core Collection (Clarivate) n=7,303**
Indexes included: SCI-EXPANDED (1945 – present), SSCI (1956 – present), AHCI (1975 – present), ESCI (2020 - present)
Date of search: January 10, 2025
Search Mode: Exact search

|  | **Query** | **Results** |
| --- | --- | --- |
| S1 | TS=(apraxia OR "atypical brain development" OR "clumsy child*" OR damp OR dcd OR "deficits in attention motor control and perception" OR "developmental coordination disorder*" OR "developmental motor coordination disorder" OR dyscoordination OR dyspraxia OR in-coordination OR "lower motor competence*" OR "mild motor problem*" OR "minimal brain damage*" OR "minimal brain dysfunction" OR "minimal cerebral dysfunction" OR "minimal neurological dysfunction" OR "minor neurological dysfunction" OR "motor coordination difficult*" OR "motor coordination problem*" OR "motor delay disorder*" OR "motor learning difficult*" OR "motor skills disorder*" OR "movement difficult*" OR "neurodevelopmental disorder*" OR "non-verbal learning disorder*" OR "perceptual motor dysfunction*" OR "perceptual motor impairment" OR "physical* awkward*" OR "psychomotor disorder*" OR "sensory integrative disorder*" OR "sensory motor difficult*" OR spddmf OR "specific developmental disorder of motor function*" ) | 63,728 |
| S2 | TS=("active video gam*" OR "attention* focus" OR avg OR "cognitive orientation to daily occupational performance*" OR co-op OR "CO OP" OR danc* OR exercise* OR "handwriting teaching" OR imagery OR intervention* OR karate OR "motivation* learning" OR "motivation* coaching" OR "motor learning" OR "motor performance" OR ot OR pa OR pe OR "physical activit*" OR "physical education" OR physiotherap* OR play* OR rehabilit* OR sport* OR therap* OR train* OR treatment* OR yoga) | 15,962,153 |
| S3 | TS=(adolescen* OR child* OR kid* OR school-age* OR "school age*" OR teenag* OR "young adult*" OR youth ) | 3,960,243 |
| S4 | S1 AND S2 AND S3 | 8,618 |
| S5 | Limit to: Publication Years: 2012- | 7,303 |

**Grey literature**

Additional studies were identified using Google Scholar and the selected search terms: dcd or ”developmental coordination disorder” in combination with any of the terms: treatment, therapy, training, or intervention. Publication limit 2012 an onward was used.

## Reference List S1

## Reference List of included Studies

Adaikina, A. (2017). *Comparing the effect of task-oriented intervention program vs. Strength training program in improving motor proficiency in children aged 8-12 years with developmental coordination disorder (dcd): A randomized controlled pilot study* [Master’s thesis, Waikato Institute of Technology]. New Zealand.

Adams, I. L. J., Smits-Engelsman, B., Lust, J. M., Wilson, P. H., & Steenbergen, B. (2017). Feasibility of motor imagery training for children with developmental coordination disorder - a pilot study. *Frontiers in Psychology*, *8*, 1271. https://doi.org/doi:10.3389/fpsyg.2017.01271

Adi-Japha, E., & Brestel, G. (2020). Motor skill learning with impaired transfer by children with developmental coordination disorder. *Research in Developmental Disabilities*, *103*, 103671. https://doi.org/10.1016/j.ridd.2020.103671

Al-Yahya, E., Esser, P., Weedon, B. D., Joshi, S., Liu, Y. C., Springett, D. N., Salvan, P., Meaney, A., Collett, J., Inacio, M., Delextrat, A., Kemp, S., Ward, T., Izadi, H., Johansen-Berg, H., Ayaz, H., & Dawes, H. (2023). Motor learning in developmental coordination disorder: Behavioral and neuroimaging study. *Frontiers in Neuroscience*, *17*, 1187790. https://doi.org/10.3389/fnins.2023.1187790

Amini, H., & Jabri, M. (2015). The effect of gymnastics on the neuropsychological characteristics of children with DCD. *Growth and Motor Learning Magazine*, *7*(2), 2–10.

Anderson, L., Wilson, J., & Carmichael, K. (2018). Implementing the cognitive orientation to daily occupational performance (co-op) approach in a group format with children living with motor coordination difficulties. *Australian Occupational Therapy Journal*, *65*(4), 295–305. https://doi.org/10.1111/1440-1630.12479

Araujo, C. R. S., Cardoso, A. A., & Magalhaes, L. D. (2019). Efficacy of the cognitive orientation to daily occupational performance with Brazilian children with developmental coordination disorder. *Scandinavian Journal of Occupational Therapy*, *26*(1), 46–54. https://doi.org/10.1080/11038128.2017.1417476

Araujo, C. R. S., Cardoso, A. A., Polatajko, H. J., & de Castro Magalhães, L. (2021). Efficacy of the cognitive orientation to daily occupational performance (co-op) approach with and without parental coaching on activity and participation for children with developmental coordination disorder: A randomized clinical trial. *Research in Developmental Disabilities*, *110*, 103862. https://doi.org/10.1016/j.ridd.2021.103862

Arramon, H., Blanchet, M., Maïano, C., & Cadoret, G. (2023). Trouble développemental de la coordination et schéma corporel: L’entraînement du couplage perception-action en intervention psychomotrice. *Neuropsychiatrie de l'Enfance et de l'Adolescence*, *71*(8), 389–398. https://doi.org/10.1016/j.neurenf.2023.05.005

Asaseh, M., Azar, J. H., & Pishyare, E. (2016). The effect of motor imagery on the gross motor skills of children with developmental coordination disorder. *Bulletin de la Société Royale des Sciences de Liège*, *85*, 130–139.

Ashkenazi, T., Laufer, Y., Ashkenazi, T., Orian, D., & Weiss, P. L. (2013). *Effect of training children with developmental coordination disorders in a virtual environment compared with a conventional environment* [Conference paper]. International Conference on Virtual Rehabilitation (ICVR), Philadelphia, PA, USA.

Au, M. K., Chan, W. M., Lee, L., Chen, T. M., Chau, R. M., & Pang, M. Y. (2014). Core stability exercise is as effective as task-oriented motor training in improving motor proficiency in children with developmental coordination disorder: A randomized controlled pilot study. *Clinical Rehabilitation*, *28*(10), 992–1003. https://doi.org/10.1177/0269215514527596

Austermann, G. (2014). How do I tell the child. The influence of instructions on acquisition of ball skills in children with development coordination disorder. *Ergotherapie Rehab*, *53*(1), 32–35.

Avalos-Robles, K. F., Viñals-Labañino, C. P., Arellano-Saldaña, E., & Arenas-Sordo, M. L. (2024). Developmental coordination disorder: Comparison between 2 therapeutic models. *Clinical Pediatrics*, *64*(2), 183–186. https://doi.org/10.1177/00099228241258608

Bahram, A., Alizade, H., Ghadiri, F., & Gheitasi, M. (2024). The effect of 8 weeks of exergame training on motor proficiency of children and adolescents with typical development and those with developmental coordination disorder. *Scientific Journal of Rehabilitation Medicine*, *13*(1) 224–241. https://doi.org/10.32598/sjrm.13.1.3

Bardid, F., Deconinck, F. J., Descamps, S., Verhoeven, L., De Pooter, G., Lenoir, M., & D'Hondt, E. (2013). The effectiveness of a fundamental motor skill intervention in pre-schoolers with motor problems depends on gender but not environmental context. *Research in Developmental Disabilities*, *34*(12), 4571–4581. https://doi.org/10.1016/j.ridd.2013.09.035

Bartov, R., Wagner, M., Shvalb, N., & Hochhauser, M. (2023). Enhancing handwriting performance of children with developmental coordination disorder (DCD) using computerized visual feedback. *Children*, *10*(9), 1534. https://doi.org/10.3390/children10091534

Basdekidou, C., Styliadis, A., Argyriadis, A., & Dimen, L. (2024). Use of geometry and featural cues in landmark configurations to reorient dcd children to the VR space: A route-learning study. *Revista de Investigacion e Innovacion en Ciencias de la Salud*, *6*(2), 5–39. https://doi.org/10.46634/riics.263

Beltrame, T. S., Cardoso, F. L., Alexandre, J. M., & Bernardi, C. S. (2016). Motor development and self-concept of children with developmental coordination disorder. *Psicologia Escolar e Educacional*, *20*(1), 55–67. https://doi.org/10.1590/2175-353920150201925

Berg, A., & Solli Fitzgerald, L. (2013). Neuro-physiological training for children with motor difficulties. *Fysioterapeuten*, *80*(5), 20–24.

Bhayanil, K., & Singaravelan, R. M. (2012). Effectiveness of core stability training programme on improving task specific physical activity in developmental coordination disorder children. *Romanian Journal of Physical Therapy / Revista Romana de Kinetoterapie*, *18*(30), 33–40.

Bonney, E., Ferguson, G., & Smits-Engelsman, B. (2017). The efficacy of two activity-based interventions in adolescents with developmental coordination disorder. *Research in Developmental Disabilities*, *71*, 223–236. https://doi.org/doi:10.1016/j.ridd.2017.10.013

Bonney, E., Jelsma, D., Ferguson, G., & Smits-Engelsman, B. (2017). Variable training does not lead to better motor learning compared to repetitive training in children with and without DCD when exposed to active video games. *Research in Developmental Disabilities*, *62*, 124–136. https://doi.org/10.1016/j.ridd.2017.01.013

Bonney, E., Jelsma, L. D., Ferguson, G. D., & Smits-Engelsman, B. C. M. (2017). Learning better by repetition or variation? Is transfer at odds with task specific training? *PLoS ONE*, *12*(3), e0174214. https://doi.org/10.1371/journal.pone.0174214

Bonney, E., Rameckers, E., Ferguson, G., & Smits-Engelsman, B. (2018). "Not just another Wii training": A graded Wii protocol to increase physical fitness in adolescent girls with probable developmental coordination disorder-a pilot study. *BMC Pediatrics*, *18*(1), 78. https://doi.org/10.1186/s12887-018-1029-7

Bortone, I., Barsotti, M., Leonardis, D., Crecchi, A., Tozzini, A., Bonfiglio, L., & Frisoli, A. (2020). Immersive virtual environments and wearable haptic devices in rehabilitation of children with neuromotor impairments: A single-blind randomized controlled crossover pilot study. *Journal of neuroengineering and rehabilitation*, *17*(1), 144. https://doi.org/10.1186/s12984-020-00771-6

Braaksma, P., Stuive, I., Jelsma, D., Van der Sluis, C. K., Dekker, R., & Schoemaker, M. M. (2022). Effectiveness and feasibility of we12bfit!: Improving physical fitness and lifestyle physical activity in children with developmental coordination disorder in a paediatric rehabilitation setting-a small sample field study. *BMJ Open*, *12*(4), e044626. https://doi.org/10.1136/bmjopen-2020-044626

Caçola, P., Romero, M., Ibana, M., & Chuang, J. (2016). Effects of two distinct group motor skill interventions in psychological and motor skills of children with developmental coordination disorder: A pilot study. *Disability and Health Journal*, *9*(1), 172–178. https://doi.org/10.1016/j.dhjo.2015.07.007

Caçola, P. M., Ibana, M., Romero, M., & Chuang, J. (2016). The effectiveness of a group motor skill intervention program in children with developmental coordination disorder: Program frequency matters. *Internet Journal of Allied Health Sciences & Practice*, *14*(1), 1–8. https//doi.org/10.46743/1540-580X/2016.1567

Cândido Soares, J. C., Costa de Moraes, B. L., dos Santos Couto Paz, C. C., & Magalhães, L. d. C. (2019). Influence of the Microsoft Kinect® games on the motor and functional performance of a child with developmental coordination disorder. *Brazilian Journal of Occupational Therapy / Cadernos Brasileiros de Terapia Ocupacional*, *27*(4), 710–717. https://doi.org/10.4322/2526-8910.ctoAO1630

Capistran, J., & Martini, R. (2016). Exploring inter-task transfer following a co-op approach with four children with DCD: A single subject multiple baseline design. *Human Movement Science*, *49*, 277–290. https://doi.org/10.1016/j.humov.2016.07.004

Carvalho, L. S. d., Crancianinov, C. S. A., Gama, D. T., & Hiraga, C. Y. (2020). Effect of volume of practice in children with probable developmental coordination disorder. *Revista Brasileira de Cineantropometria & Desempenho Humano*, *22*, 1–10. https://doi.org/10.1590/1980-0037.2020v22e72028

Cavalcante Neto, J. L., Steenbergen, B., Wilson, P., Zamunér, A. R., & Tudella, E. (2020). Is Wii-based motor training better than task-specific matched training for children with developmental coordination disorder? A randomized controlled trial. *Disability and Rehabilitation*, *42*(18), 2611–2620. https://doi.org/10.1080/09638288.2019.1572794

Cavalcante Neto, J. L., Steenbergen, B., Zamunér, A. R., & Tudella, E. (2021). Wii training versus non-Wii task-specific training on motor learning in children with developmental coordination disorder: A randomized controlled trial. *Annals of Physical and Rehabilitation Medicine*, *64*(2), 101390. https://doi.org/10.1016/j.rehab.2020.03.013

Cavalcante Neto, J. L., Zamunér, A. R., Silva, R. A. d. S., Menegat, D., Silva, E., & Tudella, E. (2017). Effects of motor skills training program on the cardiac autonomic control in children with developmental coordination disorder: A preliminary study. *European Journal of Physiotherapy*, *19*, 56–58. https://doi.org/10.1080/21679169.2017.1381314

Cavalcante-Neto, J. L., Jelsma, D., Draghi, T. T. G., Tudella, E., & Smits-Engelsman, B. (2022). Active video games performance and heart rate on the Wii or Kinect in children with and without developmental coordination disorder. *Children*, *9*(12), 1823. https://doi.org/10.3390/children9121823

Cheng, Y. T. Y., Wong, T. K. S., Tsang, W. W. N., Schooling, C. M., Fong, S. S. M., Fong, D. Y. T., Gao, Y., & Chung, J. W. Y. (2019). Neuromuscular training for children with developmental coordination disorder: A randomized controlled trial. *Medicine*, *98*(45), e17946. https://doi.org/10.1097/MD.0000000000017946

Coetzee, D., & Pienaar, A. E. (2013). The effect of visual therapy on the ocular motor control of seven- to eight-year-old children with developmental coordination disorder (DCD). *Research in Developmental Disabilities*, *34*(11), 4073–4084. https://doi.org/10.1016/j.ridd.2013.08.036

Condomines, E., & Lewandowski, C. F. (2024). Neuromotor task training (NTT) in the psychomotor rehabilitation of developmental coordination disorder (DCD): A clinical case study on buttoning with an 8-year-old child. *ANAE - Approche Neuropsychologique des Apprentissages chez l'Enfant*, *36*(191), 439–449.

Dannenbaum, E., Bégin, C.-L., Daigneault-Bourgeois, É., Kwon Pak Yin, N., Laferrière-Trudeau, C., Mazer, B., Moreau, V., Salvo, L., Villeneuve, M., & Lamontagne, A. (2021). Feasibility and preliminary effects of a 1-week vestibular rehabilitation day camp in children with developmental coordination disorder. *Physical & Occupational Therapy in Pediatrics*, *42*(1), 62–79. https://doi.org/10.1080/01942638.2021.1925800

de Medeiros, P., Libardoni dos Santos, J. O., Capistrano, R., Carvalho, H. P., Beltrame, T. S., & Cardoso, F. L. (2018). Effects of exergames in children at risk and significant movement difficulty: A blinded randomized study. *Revista Brasileira de Ciencias do Esporte*, *40*(1), 87–93. https://doi.org/10.1016/j.rbce.2018.01.005

De Milander, M., Coetzee, F. F., & Venter, A. (2015). Perceptual-motor intervention for developmental coordination disorder in grade 1 children. *South African Journal for Research in Sport Physical Education and Recreation*, *37*(2), 15–32. https://hdl.handle.net/10520/EJC177833

De Milander, M., Du Plessis, J., & Du Randt, A. (2014). Sport stacking motor intervention programme for children with developmental coordination disorder. *South African Journal for Research in Sport Physical Education and Recreation*, *36*(3), 51–60.

de Oliveira, I. S., Spessato, B. C., da Silva Oliveira, D., Lins, R. J. C., & Sarinho, S. W. (2022). Long-term effects of a movement intervention program for children with probable developmental coordination disorder. *Journal of Teaching in Physical Education*, *41*(4), 680–688. https://doi.org/10.1123/jtpe.2021-0124

Diaz-Perez, A., Vicente-Nicolas, G., & Valero-Garcia, A. V. (2020). Music, body movement, and dance intervention program for children with developmental coordination disorder. *Psychology of Music*, *49*(5), 1215–1225. https://doi.org/10.1177/0305735620936353

Draghi, T. T. G., Smits-Engelsman, B., Godoi-Jacomassi, D., Cavalcante Neto, J. L., Jelsma, D., & Tudella, E. (2024). Short- and long-term changes in balance after active video game training in children with and without developmental coordination disorder: A randomized controlled trial. *Motor Control*, *28*(2), 174–192. https://doi.org/10.1123/mc.2023-0070

EbrahimiSani, S., Sohrabi, M., Taheri, H., Agdasi, M. T., & Amiri, S. (2020). Effects of virtual reality training intervention on predictive motor control of children with DCD - a randomized controlled trial. *Research in Developmental Disabilities*, *107*, 103768. https://doi.org/10.1016/j.ridd.2020.103768

Eslamdost, M., Sheikh, M., Mohammadi, M., & Ahmadi, G. (2017). The effect of core stability training on static and dynamic balance in children with developmental coordination disorder. *Community Health Journal*, *11*(2), 20–28. https://doi.org/10.22123/chj.2018.85447

Farhat, F., Hsairi, I., Baati, H., Smits-Engelsman, B. C. M., Masmoudi, K., McHirgui, R., Triki, C., & Moalla, W. (2016). The effect of a motor skills training program in the improvement of practiced and non-practiced tasks performance in children with developmental coordination disorder (DCD). *Human Movement Science*, *46*, 10–22. https://doi.org/10.1016/j.humov.2015.12.001

Farhat, F., Masmoudi, K., Hsairi, I., Smits-Engelsman, B. C. M., McHirgui, R., Triki, C., & Moalla, W. (2015). The effects of 8 weeks of motor skill training on cardiorespiratory fitness and endurance performance in children with developmental coordination disorder. *Applied physiology, nutrition, and metabolism*, *40*(12), 1269–1278. https://doi.org/10.1139/apnm-2015-0154

Ferguson, G. D., Jelsma, D., Jelsma, J., & Smits-Engelsman, B. C. M. (2013). The efficacy of two task-orientated interventions for children with developmental coordination disorder: Neuromotor task training and Nintendo Wii Fit training. *Research in Developmental Disabilities*, *34*(9), 2449–2461. https://doi.org/10.1016/j.ridd.2013.05.007

Ferguson, G. D., Naidoo, N., & Smits-Engelsman, B. C. M. (2015). Health promotion in a low-income primary school: Children with and without DCD benefit, but differently. *Physical & Occupational Therapy in Pediatrics*, *35*(2), 147–162. https://doi.org/10.3109/01942638.2015.1009230

Fong, S. M. (2012). *Effect of taekwondo training on sensori-motor performance and postural control in children with and without developmental coordination disorder* [Thesis dissertation Hong Kong Polytechnic University]. Hong Kong. https://search.ebscohost.com/login.aspx?direct=true&AuthType=cookie,ip,uid&db=c8h&AN=109860247&site=ehost-live

Fong, S. S., Chung, J. W., Chow, L. P., Ma, A. W., & Tsang, W. W. (2013). Differential effect of taekwondo training on knee muscle strength and reactive and static balance control in children with developmental coordination disorder: A randomized controlled trial. *Research in Developmental Disabilities*, *34*(5), 1446–1455. https://doi.org/10.1016/j.ridd.2013.01.025

Fong, S. S., Tsang, W. W., & Ng, G. Y. (2012). Taekwondo training improves sensory organization and balance control in children with developmental coordination disorder: A randomized controlled trial. *Research in Developmental Disabilities*, *33*(1), 85–95. https://doi.org/10.1016/j.ridd.2011.08.023

Fong, S. S. M., Chung, L. M. Y., Schooling, C. M., Lau, E. H. Y., Wong, J. Y. H., Bae, Y.-H., & Chung, J. W. Y. (2022). Tai chi-muscle power training for children with developmental coordination disorder: A randomized controlled trial. *Scientific Reports*, *12*(1), 22078. https://doi.org/10.1038/s41598-022-25822-x

Fong, S. S. M., Guo, X., Cheng, Y. T. Y., Liu, K. P. Y., Tsang, W. W. N., Yam, T. T. T., Chung, L. M. Y., & Macfarlane, D. J. (2016). A novel balance training program for children with developmental coordination disorder: A randomized controlled trial. *Medicine*, *95*(16), e3492. https://doi.org/10.1097/MD.0000000000003492

Fong, S. S. M., Guo, X., Liu, K. P. Y., Ki, W. Y., Louie, L. H. T., Chung, R. C. K., & Macfarlane, D. J. (2016). Task-specific balance training improves the sensory organisation of balance control in children with developmental coordination disorder: A randomised controlled trial. *Scientific Reports*, *6*, 20945. https://doi.org/10.1038/srep20945

Fort, K. M., & Overton, K. J. (2015). *Nintendo Wii Fit (TM) intervention for balance improvement in a child with developmental coordination disorder* [Doctoral dissertation, Florida Gulf Coast University].

Franceschini, S., Bertoni, S., Lulli, M., Pievani, T., & Facoetti, A. (2022). Short-term effects of video-games on cognitive enhancement: The role of positive emotions. *Journal of Cognitive Enhancement*, *6*(1), 29–46. https://doi.org/10.1007/s41465-021-00220-9

Ganapathy Sankar, U., & Monisha, R. (2020). A core stability program for a child with developmental coordination disorder: Single case study. *International Journal of Pharmaceutical Research*, *12*, 1958–1961. https://doi.org/10.31838/ijpr/2020.SP2.346

Ganapathy Sankar, U., & Monisha, R. (2020). Effectiveness of motor imagery training for children with developmental coordination disorder among Indian children - a pilot study. *International Journal of Research in Pharmaceutical Sciences*, *11*(4), 6522–6525. https://doi.org/10.26452/ijrps.v11i4.3534

Ganapathy Sankar, U., & Monisha, R. (2020). Effectiveness of visual memory training for primary school children with developmental coordination disorder (DCD). *International Journal of Research in Pharmaceutical Sciences*, *11*(4), 6206–6210. https://doi.org/10.26452/ijrps.v11i4.3297

Ganapathy Sankar, U., & Monisha, R. (2020). Effects of neuromotor task training (NTT) - a new approach for children with developmental coordination disorder (DCD) in Indian context. *International Journal of Research in Pharmaceutical Sciences*, *11*(4), 6459–6462. https://doi.org/10.26452/ijrps.v11i4.3444

Ganapathy Sankar, U., & Monisha, R. (2020). Efficacy of the cognitive orientation to daily occupational performance in Indian children with developmental coordination disorder. *International Journal of Research in Pharmaceutical Sciences*, *11*(4), 6473–6477. https://doi.org/10.26452/ijrps.v11i4.3463

Ganapathy, S. U., & Monisha, R. (2020). Effectiveness of two task-oriented interventions over cardiorespiratory fitness and motor performance in children with developmental coordination disorder (DCD) - a pilot study. *International Journal of Research in Pharmaceutical Sciences*, *11*(4), 6399–6403. https://doi.org/10.26452/ijrps.v11i4.3400

Gazya, A., Sherief, A. A. A., Abd El Ghaffar, M. A., & Aneis, Y. M. (2014). Effect of isokinetic training on knee muscle strength and balance control in children with developmental coordination disorder: A randomized controlled trial. *Medical Journal of Cairo University, 82*(2), 69-78.

Ghadiri, F., O'Brien, W., Soltani, S., Faraji, M., & Bahmani, M. (2022). The effect of karate interventions on the motor proficiency of female adolescents with developmental coordination disorder (DCD) from high and low socio-economic status. *BMC Sports Science Medicine and Rehabilitation*, *14*(1), 121. https://doi.org/10.1186/s13102-022-00501-6

Gholami, A., & Sabzi, A. H. (2021). The effect of perceptual-motor and vestibular training on static and dynamic balance in children with developmental coordination disorder. *Student Sports & Health Open Researches e-journal: New-approaches*, *1*(1).

Ghorbanzadeh, B., Kirazci, S., & Badicu, G. (2024). Comparison of the effect of teaching games for understanding, sport education, combined and linear pedagogy on motor proficiency of children with developmental coordination disorder. *Frontiers in Psychology*, *15*, 1385289. https://doi.org/10.3389/fpsyg.2024.1385289

Giagazoglou, P., Sidiropoulou, M., Mitsiou, M., Arabatzi, F., & Kellis, E. (2015). Can balance trampoline training promote motor coordination and balance performance in children with developmental coordination disorder? *Research in Developmental Disabilities*, *36*, 13–19. https://doi.org/10.1016/j.ridd.2014.09.010

Gill, K. K., Lang, D., & Zwicker, J. G. (2022). Cerebellar differences after rehabilitation in children with developmental coordination disorder. *Brain Sciences*, *12*(7), 856. https://doi.org/10.3390/brainsci12070856

Grohs, M. N., Craig, B. T., Kirton, A., & Dewey, D. (2020). Effects of transcranial direct current stimulation on motor function in children 8–12 years with developmental coordination disorder: A randomized controlled trial. *Frontiers in Human Neuroscience*, *14*, 608131. https://doi.org/10.3389/fnhum.2020.608131

Hammond, J., Jones, V., Hill, E. L., Green, D., & Male, I. (2014). An investigation of the impact of regular use of the Wii Fit to improve motor and psychosocial outcomes in children with movement difficulties: A pilot study. *Child: Care, Health and Development*, *40*(2), 165–175. https://doi.org/10.1111/cch.12029

Hands, B., Chivers, P., Grace, T., & McIntyre, F. (2019). Time for change: Fitness and strength can be improved and sustained in adolescents with low motor competence. *Research in Developmental Disabilities*, *84*, 131–138. https://doi.org/10.1016/j.ridd.2018.07.009

Harkness-Armstrong, C., Hodson-Tole, E., Wood, G., & Mills, R. (2025). Short report on a 6-week at-home exergaming intervention to improve balance in children with developmental coordination disorder. *Research in Developmental Disabilities*, *156*, 104900. https://doi.org/10.1016/j.ridd.2024.104900

Hartmann, P., & Schönthaler, E. (2020). Generalisierung und transfer von fertigkeiten und strategien nach therapie mit dem co-op-ansatz bei kindern mit einer umschriebenen entwicklungsstörung der motorischen funktionen (uemf) - eine mixed-method studie. *Ergoscience*, *15*(4), 152–162. https://doi.org/10.2443/skv-s-2020-54020200403

Hashemi, A., & Shahrbanian, S. (2017). The effects of sensorimotor integration exercises on gross motor skills of children with motor coordination disorder. https://www.researchgate.net/publication/361209206

Hashemi, A., Khodaverdi, Z., & Zamani, M. H. (2022). Effect of Wii Fit training on visual perception and executive function in boys with developmental coordination disorders: A randomized controlled trial. *Research in Developmental Disabilities*, *124*, 104196. https://doi.org/10.1016/j.ridd.2022.104196

Hashemi, A., Sheikh, M., & Hemayattalab, R. (2016). The effects of regular exercise on neuropsychological characteristics of the children with developmental coordination disorder. *Journal of Psychological Science*, *15*(58), 261–275.

Hashemi, A., Sheikh, M., Homaneyan, D., & Bagherzaeh, F. (2019). The effect of wii fit training on metacognitive characteristics of children with developmental coordination disorder. *Journal of Motor and Behavioral Sciences*, *2*(3), 177–188.

Hatanaka, R., Higuchi, Y., & Imaoka, M. (2024). Improving motor skills in five children with developmental coordination disorder traits and its impact on parenting stress: A case series. *Cureus*, *16*(6), e61691. https://doi.org/10.7759/cureus.61691

Hession, C. E., Eastwood, B., Watterson, D., Lehane, C. M., OxIey, N., & Murphy, B. A. (2014). Therapeutic horse riding improves cognition, mood arousal, and ambulation in children with dyspraxia. *Journal of Alternative and Complementary Medicine*, *20*(1), 19–23. https://doi.org/10.1089/acm.2013.0207

Hession, C. E., Law Smith, M. J., Watterson, D., Oxley, N., & Murphy, B. A. (2019). The impact of equine therapy and an audio-visual approach emphasizing rhythm and beat perception in children with developmental coordination disorder. *Journal of Alternative and Complementary Medicine*, *25*(5), 535–541. https://doi.org/10.1089/acm.2017.0242

Heus, I., Weezenberg, D., Severijnen, S., Vliet Vlieland, T., & van der Holst, M. (2022). Measuring treatment outcome in children with developmental coordination disorder; responsiveness of six outcome measures. *Disability and Rehabilitation*, *44*(7), 1023–1034. https://doi.org/10.1080/09638288.2020.1785022

Howie, E. K., Campbell, A. C., Abbott, R. A., & Straker, L. M. (2017). Understanding why an active video game intervention did not improve motor skill and physical activity in children with developmental coordination disorder: A quantity or quality issue? *Research in Developmental Disabilities*, *60*, 1–12. https://doi.org/10.1016/j.ridd.2016.10.013

Howie, E. K., Campbell, A. C., & Straker, L. M. (2016). An active video game intervention does not improve physical activity and sedentary time of children at-risk for developmental coordination disorder: A crossover randomized trial. *Child: Care, Health and Development*, *42*(2), 253–260. https://doi.org/10.1111/cch.12305

Hurschler Lichtsteiner, S., Nideröst, M., Di Brina, C., Marquardt, C., Wyss, S., Buholzer, A., & Wicki, W. (2023). Effectiveness of psychomotor therapy among children with graphomotor impairment with and without DCD-diagnosis. *Children*, *10*(6), 964. https://doi.org/10.3390/children10060964

Hyland, M., & Polatajko, H. J. (2012). Enabling children with developmental coordination disorder to self-regulate through the use of dynamic performance analysis: Evidence from the co-op approach. *Human Movement Science*, *31*(4), 987–998. https://doi.org/10.1016/j.humov.2011.09.003

Imai, Y., Naruse, H., Kurita, R., Hiratani, M., & Tomori, K. (2020). A hybrid intervention combining group-based physical activity and an individual goal-oriented approach for children with developmental disabilities: A pre-post intervention study. *International Journal of Disability, Development & Education*, *70*(1), 1–17. https://doi.org/10.1080/1034912X.2020.1856793

Inacio, M., Esser, P., Weedon, B. D., Joshi, S., Meaney, A., Delextrat, A., Springett, D., Kemp, S., Ward, T., Izadi, H., Johansen-Berg, H., & Dawes, H. (2023). Learning a novel rhythmic stepping task in children with probable developmental coordination disorder. *Clinical Biomechanics*, *102*, 105904. https://doi.org/10.1016/j.clinbiomech.2023.105904

Izadi-Najafabadi, S., Gunton, C., Dureno, Z., & Zwicker, J. G. (2022). Effectiveness of cognitive orientation to occupational performance intervention in improving motor skills of children with developmental coordination disorder: A randomized waitlist-control trial. *Clinical Rehabilitation*, *36*(6), 776–788. https://doi.org/10.1177/02692155221086188

Izadi-Najafabadi, S., Rinat, S., & Zwicker, J. G. (2022). Brain functional connectivity in children with developmental coordination disorder following rehabilitation intervention. *Pediatric Research*, *91*(6), 1459–1468. https://doi.org/10.1038/s41390-021-01517-3

Izadi-Najafabadi, S., & Zwicker, J. G. (2021). White matter changes with rehabilitation in children with developmental coordination disorder: A randomized controlled trial. *Frontiers in Human Neuroscience*, *15*, 673003. https://doi.org/10.3389/fnhum.2021.673003

Jahanbakhsh, H., Sohrabi, M., Kakhki, A. S., & Khodashenas, E. (2020). The effect of task-specific balance training program in dual-task and single-task conditions on balance performance in children with developmental coordination disorder. *Acta Gymnica*, *50*(1), 28–37. https://doi.org/10.5507/ag.2020.003

Jalilvand, M., Samadi, H., & Heydari, Y. (2021). The effects of executive function training program by physical activity on response inhibition in children with developmental coordination disorder. *Middle Eastern Journal of Disability Studies*, *11*(1).

Jelsma, D., Geuze, R. H., Mombarg, R., & Smits-Engelsman, B. C. M. (2014). The impact of Wii Fit intervention on dynamic balance control in children with probable developmental coordination disorder and balance problems. *Human Movement Science*, *33*, 404–418. https://doi.org/10.1016/j.humov.2013.12.007

Jelsma, D., Targino Gomes Draghi, T., Cavalcante Neto, J., & Smits-Engelsman, B. (2023). Improved attentional abilities after playing five weeks of active video games in children with and without developmental coordination disorder. *Applied Neuropsychology: Child*, *13*(4), 350–358. https://doi.org/10.1080/21622965.2023.2190024

Jelsma, L. D., Cavalcante Neto, J. L., Smits-Engelsman, B., Targino Gomes Draghi, T., Araújo Rohr, L., & Tudella, E. (2023). Type of active video-games training does not impact the effect on balance and agility in children with and without developmental coordination disorder: A randomized comparator-controlled trial. *Applied Neuropsychology: Child*, *12*(1), 64–73. https://doi.org/10.1080/21622965.2022.2030740

Jelsma, L. D., Geuze, R. H., & Smits-Engelsman, B. C. M. (2020). Movement control strategies in a dynamic balance task in children with and without developmental coordination disorder. *Journal of Motor Behavior*, *52*(2), 175–186. https://doi.org/10.1080/00222895.2019.1599809

Jie, Y., Sit, C. H. P., Burnett, A., Capio, C. M., Ha, A. S. C., & Huang, W. Y. J. (2016). Effects of fundamental movement skills training on children with developmental coordination disorder. *Adapted Physical Activity Quarterly*, *33*(2), 134–155. https://doi.org/10.1123/APAQ.2015-0008

Jokar Tang Karami, S., Sheikh, M., & Bagherzadeh, F. (2018). The effect of a period of selected physical activity on improving gross motor skills in children with developmental coordination disorder (DCD). *Journal of Sports and Motor Development and Learning*, *10*(1), 23–36. https://doi.org/10.22059/jmlm.2017.29136

Jokić, C. S., Polatajko, H., & Whitebread, D. (2013). Self-regulation as a mediator in motor learning: The effect of the cognitive orientation to occupational performance approach on children with DCD. *Adapted Physical Activity Quarterly*, *30*(2), 103–126. https://doi.org/10.1123/apaq.30.2.103

Jokić, C. S., & Whitebread, D. (2014). Examining change in metacognitive knowledge and metacognitive control during motor learning: What can be learned by combining methodological approaches? *Psihologijske Teme*, *23*(1), 53–76.

Ju, Y. J., Du, Y. C., Huang, H. C., Hu Kao, P. S., & Cherng, R. J. (2023). Development and feasibility of a virtual reality-based exergaming program to enhance cardiopulmonary fitness in children with developmental coordination disorder. *Frontiers in Pediatrics*, *11*, 1238471. https://doi.org/10.3389/fped.2023.1238471

Ju, Y. J., Du, Y. C., Lin, L. Y., Hou, C. R., Lin, P. Y., & Cherng, R. J. (2018). The effect of laboratory-developed video games on balance performance in children with developmental coordination disorder. *Biomedical Engineering-Applications Basis Communications*, *30*(1), 8. https://doi.org/10.4015/s1016237218500059

Kaiser, M. L. (2013). Children with developmental coordination disorder: The effects of combined intervention on motor coordination, occupational performance, and quality of life. *Journal of Occupational Therapy, Schools, and Early Intervention*, *6*(1), 44–53. https://doi.org/10.1080/19411243.2013.771100

Kalkhoran, J. F., Amini, H. A., Salman, Z., & Zareiyan, E. (2018). Gymnastics training improves executive and sensorimotor functions in children with developmental coordination disorder. *Journal of Integrative Pediatric Healthcare*, *2*(1), 2–6.

Kangarani-Farahani, M., Thompson-Hodgetts, S., & Zwicker, J. G. (2025). Effectiveness of cognitive orientation to daily occupational performance for autistic children with developmental coordination disorder. *Developmental Medicine and Child Neurology*, *67*(2), 216–222. https://doi.org/10.1111/dmcn.16058

Kennedy-Behr, A., Rodger, S., Graham, F., & Mickan, S. (2013). Creating enabling environments at preschool for children with developmental coordination disorder. *Journal of Occupational Therapy, Schools, and Early Intervention*, *6*(4), 301–313. https://doi.org/10.1080/19411243.2013.860760

Khanjani, M., Bagherli, J., Nasiri, I., & Namazizadeh, M. (2021). Comparing participatory and competitive games effect on self-efficacy in primary school girls with developmental coordination disorder. *Middle Eastern* *Journal of Disability Studies*, *11*(1), 1-19.

Khanjankhani, E., Samadi, H., Ahar, S., & Romero-Naranjo, F. J. (2024). The effect of bapne body percussion exercises on the balance and the executive functions of DCD children: A preliminary study. *Per Musi*, *25*, 1–19, Article e242502. https://doi.org/10.35699/2317-6377.2024.49095

Kim, D., Roh, W., Lee, Y., & Yim, S. (2024). The effect of a table tennis exercise program with a task-oriented approach on visual perception and motor performance of adolescents with developmental coordination disorder. *Perceptual & Motor Skills*, *131*(4), 1183–1206. https://doi.org/10.1177/00315125241250080

Kluwe, M., Miyahara, M., & Heveldt, K. (2012). A case study to evaluate balance training with movement test items and through teaching observation: Beyond specificity and transfer of learning. *Physical Education and Sport Pedagogy*, *17*(5), 463–475. https://doi.org/10.1080/17408989.2011.594428

Kopecká, J., & Klobucká, S. (2019). Effect of kinesiotherapy on selected aspects of motor junctions in children with specific developmental impairment of motor functions. *Rehabilitacia*, *56*(3), 247–262.

Kordi, H., Sohrabi, M., Saberi Kakhki, A., & Attarzadeh Hossini, S. R. (2016). The effect of strength training based on process approach intervention on balance of children with developmental coordination disorder. *Archivos Argentinos De Pediatria*, *114*(6), 526–533. https://doi.org/10.5546/aap.2016.eng.526

Krajenbrink, H., Lust, J., van Heeswijk, J., Aarts, P., & Steenbergen, B. (2022). Benefits of an intensive individual co-op intervention in a group setting for children with DCD. *Occupational Therapy International*, *2022*, 8209128. https://doi.org/10.1155/2022/8209128

Kuijpers, R., Smulders, E., Groen, B. E., Smits-Engelsman, B. C. M., Nijhuis-van der Sanden, M. W. G., & Weerdesteyn, V. (2022). Walking adaptability improves after treadmill training in children with developmental coordination disorder: A proof-of-concept study. *Gait & Posture*, *92*, 258–263. https://doi.org/10.1016/j.gaitpost.2021.11.038

Lee, K. (2024). Enhancing motor performance and physical fitness in children with developmental coordination disorder through fundamental motor skills exercise. *Healthcare*, *12*(21), 2142. https://doi.org/10.3390/healthcare12212142

Lee, K., & Kim, M. J. (2018). Case study: Changes of motor skill development in children with developmental coordination disorder after 12-week after-school program. *Journal of the Korean Society of Sport Psychology, 29*(3), 209–217.

Lodal, K., & Bond, C. (2017). An exploratory product evaluation of the Manchester motor skills programme. *Educational Psychology in Practice*, *33*(2), 149–166. https://doi.org/10.1080/02667363.2016.1261803

Loftesnes, J. M., Ingvaldsen, R. P., & Sigmundsson, H. (2017). Children with developmental coordination disorder. *Psychological Reports*, *120*(2), 242–254. https://doi.org/10.1177/0033294116687761

Ma, A. W. W., Fong, S. S. M., Guo, X., Liu, K. P. Y., Fong, D. Y. T., Bae, Y.-H., Yuen, L., Cheng, Y. T. Y., & Tsang, W. W. N. (2018). Adapted taekwondo training for prepubertal children with developmental coordination disorder: A randomized, controlled trial. *Scientific Reports*, *8*(1), 10330. https://doi.org/10.1038/s41598-018-28738-7

Ma, W. W. A., & Wong, H. L. J. (2020). Exergaming for children with probable developmental coordination disorder (p-dcd)/dcd. *Asian Journal of Physical Education & Recreation*, 29–38. https://doi.org/10.24112/ajper.251917

Maharaj, S. S., & Lallie, R. (2016). Does a physiotherapy programme of gross motor training influence motor function and activities of daily living in children presenting with developmental coordination disorder? *South African Journal of Physiotherapy*, *72*(1), 1–9. https://doi.org/10.4102/sajp.v72i1.304

Marshall, B., Wright, D. J., Holmes, P. S., Williams, J., & Wood, G. (2020). Combined action observation and motor imagery facilitates visuomotor adaptation in children with developmental coordination disorder. *Research in Developmental Disabilities*, *98*, 103570. https://doi.org/10.1016/j.ridd.2019.103570

Martini, R., Mandich, A., & Green, D. (2014). Implementing a modified cognitive orientation to daily occupational performance approach for use in a group format. *British Journal of Occupational Therapy*, *77*(4), 214–219. https://doi.org/10.4276/030802214X13968769798917

Martini, R., & Savard, J. (2021). Cognitive orientation to daily occupational performance (co-op): 1-week group intervention with children referred for motor coordination difficulties. *Open Journal of Occupational Therapy*, *9*(3), 1–14. https://doi.org/10.15453/2168-6408.1765

McIntyre, F., Chivers, P., Larkin, D., Rose, E., & Hands, B. (2015). Exercise can improve physical self perceptions in adolescents with low motor competence. *Human Movement Science*, *42*, 333–343. https://doi.org/10.1016/j.humov.2014.12.003

McQueen, M., Parker, A., Pascoe, M., Baldwin, P., Mancini, V., Cairney, J., Wilmut, K., & Williams, J. (2024). Investigating the feasibility and acceptability of a Facebook-delivered, parent mediated, physical activity intervention for children with developmental coordination disorder. *International Journal of Disability, Development and Education,* 1-22. https://doi.org/10.1080/1034912X.2024.2411265

Mehdinejad, M., Meshkati, Z., & Badami, R. (2021). The effect of increasing cognitive load of rhythmic games on theory of mind in children with developmental coordination disorder. *Journal of Rehabilitation Sciences and Research*, *8*(1), 19–24. https://doi.org/10.30476/JRSR.2021.82617.1032

Menz, S. M., Hatten, K., & Grant-Beuttler, M. (2013). Strength training for a child with suspected developmental coordination disorder. *Pediatric Physical Therapy*, *25*(2), 214–223. https://doi.org/10.1097/PEP.0b013e31828a2042

Miles, C. A., Wood, G., Vine, S. J., Vickers, J. N., & Wilson, M. R. (2015). Quiet eye training facilitates visuomotor coordination in children with developmental coordination disorder. *Research in Developmental Disabilities*, *40*, 31–41. https://doi.org/10.1016/j.ridd.2015.01.005

Miyahara, M., Culling, M., & Green, C. (2021). Delivering clinic-and home-based programs concurrently for children with developmental coordination disorder: A multiple case study. *Palaestra*, *35*(4), 36–40.

Mohammadi Oranghi, B., Yaali, R., & Shahrzad, N. (2018). The effect of eight weeks aerobic rhythmic exercises with music on motor proficiency, anxiety and depression in children with developmental coordination disorder. *Motor Behavior*, *9*(30), 57–70. https://doi.org/10.22089/mbj.2018.3599.1437

Mombarg, R., Jelsma, D., & Hartman, E. (2013). Effect of Wii-intervention on balance of children with poor motor performance. *Research in Developmental Disabilities*, *34*(9), 2996–3003. https://doi.org/10.1016/j.ridd.2013.06.008

Moradi, H., Khodashenas, E., Sohrabi, M., Teymoori, S., & Shayan-Noshabadi, A. (2015). The effect of Spark motor program on sensory-motor functions in children with developmental coordination disorder. *Feyz Medical Sciences Journal*, *19*(5), 391–398.

Moradi, H., & Movahedi, A. (2019). Effect of environment enrichment (SPARK perceptual-motor exercises) on the improvement of neurocognitive functions in children with developmental coordination disorder. *The Neuroscience Journal of Shefaye Khatam*, *7*(3), 23–31.

Moradi, H., Sohrabi, M., & Mones-Tusi, M. (2017). The effect of exercises with contextual interference of training on attention problems in children with developmental coordination disorder. *Journal of Shahrekord University of Medical Sciences*, *19*(5), 46–59.

Morgan-Jones, M., Knott, F., Wilcox, H., & Ashwin, C. (2019). A pilot study of fascia Bowen therapy for 8-11 year-old boys with developmental coordination disorder. *Journal of Bodywork and Movement Therapies*, *23*(3), 568–574. https://doi.org/10.1016/j.jbmt.2019.02.022

Namdar. (2016). The effect of an exercise intervention on motor proficiency 10-7 in boys with developmental coordination disorder. *Journal of sport management and motor behaviour, 11*(22), 59-68. https//doi.org/[10.22080/jsmb.2015.990](https://doi.org/10.22080/jsmb.2015.990)

Navarro-Paton, R., Martin-Ayala, J. L., Marti Gonzalez, M., Hernandez, A., & Mecias-Calvo, M. (2021). Effect of a 6-week physical education intervention on motor competence in pre-school children with developmental coordination disorder. *Journal of Clinical Medicine*, *10*(9), 12. https://doi.org/10.3390/jcm10091936

Niklasson, M., Norlander, T., Niklasson, I., & Rasmussen, P. (2017). Catching-up: Children with developmental coordination disorder compared to healthy children before and after sensorimotor therapy. *PLoS ONE*, *12*(10), e0186126. https://doi.org/10.1371/journal.pone.0186126

Noordstar, J. J., van der Net, J., Voerman, L., Helders, P. J., & Jongmans, M. J. (2017). The effect of an integrated perceived competence and motor intervention in children with developmental coordination disorder. *Research in Developmental Disabilities*, *60*, 162–175. https://doi.org/10.1016/j.ridd.2016.12.002

Norouzi Seyed Hosseini, R., Norouzi, E., & Soleymani, M. (2021). Effects of quiet eye training on performance of bimanual coordination in children with DCD. *Iranian Journal of Child Neurology*, *15*(4), 43–54. https://doi.org/10.22037/ijcn.v15i4.18926

Oswald, A., & Fridrich, A. (2014). Evaluation of the combined bottom-up- and top-down-therapy method ability, skill and task training (ASTT) with children with finemotor [sic] - and grossmotor [sic]-coordination-disorder and/or dyspraxia - pilot study with children aged 3;6 to 9;9 years old. *Ergoscience*, *9*(1), 24–33. https://doi.org/10.2443/skv-s-2014-54020140103

Pesce, C., Crova, C., Marchetti, R., Struzzolino, I., Masci, I., Vannozzi, G., & Forte, R. (2013). Searching for cognitively optimal challenge point in physical activity for children with typical and atypical motor development. *Mental Health and Physical Activity*, *6*(3), 172–180. https://doi.org/10.1016/j.mhpa.2013.07.001

Purpura, G., Di Giusto, V., Zorzi, C. F., Figliano, G., Randazzo, M., Volpicelli, V., Blonda, R., Brazzoli, E., Reina, T., Rezzonico, S., Sala, R., & Cavallini, A. (2024). Use of virtual reality in school-aged children with developmental coordination disorder: A novel approach. *Sensors*, *24*(17). https://doi.org/10.3390/s24175578

Puyjarinet, F., Madramany, P., Autexier, A., Madieu, E., Nesensohn, J., & Biotteau, M. (2022). Psychomotor intervention to improve handwriting skills in children with adhd: A single-case experimental design with direct inter-subject and systematic replications. *Neuropsychological Rehabilitation*, *33*(9), 1537–1563. https://doi.org/10.1080/09602011.2022.2114503

Rameckers, E. A. A., Crafford, R., Ferguson, G., & Smits Engelsman, B. C. M. (2023). Efficacy of a task-oriented intervention for children with a dual diagnosis of specific learning disabilities and developmental coordination disorder: A pilot study. *Children*, *10*(3). https://doi.org/10.3390/children10030415

Sabzevari, H., Arsham, S., & Parvinpor, S. (2019). Effect of rhythmic motor games on motor proficiency, educational achievement and self-esteem in children with developmentally coordination disorder. *Razi Journal of Medical Sciences*, *26*(7), 66–77.

Sabzi, A. H., Damanpak, S., & Tatari Hasan Gavyar, M. (2019). Effect of 12 sessions of vestibular stimulation exercises on the balance performance in children with developmental coordination disorder. *The Scientific Journal of Rehabilitation Medicine*, *8*(3), 1–8.

Saidmamatov, O., Raximov, Q., Rodrigues, P., & Vasconcelos, O. (2021). A ten-week motor skills training program increases motor competence in children with developmental coordination disorder. *Children*, *8*(12), 11. https://doi.org/10.3390/children8121147

Saidmamatov, O., Sanjarbek, K., Vasconcelos, O., & Rodrigues, P. (2022). Sustainability of a given ten-week motor skills training program for children with developmental coordination disorder. *Sports*, *10*(11), 9. https://doi.org/10.3390/sports10110164

Saidmamatov, O. A., Nascimento, M. M., Cerqueira, J. C., Rodrigues, P., & Vasconcelos, O. (2022). Motor skill training programs for children with developmental coordination disorder: Does gender matter? *Neuropsychiatrie de l'Enfance et de l'Adolescence*, *70*(4), 183–194. https://doi.org/10.1016/j.neurenf.2022.03.001

Sánchez-Matas, Y., Gutiérrez, D., Hernández-Martínez, A., & Segovia, Y. (2022). Effect of a motor reinforcement programme in students with motor and/or social difficulties. *Cuadernos de Psicologia del Deporte*, *22*(1), 14–27. https://doi.org/10.6018/CPD.421831

Sánchez-Matas, Y., Hernández-Martínez, A., Gutiérrez, D., & Rudd, J. (2024). Actual and perceived motor competence in children with motor coordination difficulties: Effect of a movement-based intervention. *Research in Developmental Disabilities*, *151*, 104797. https://doi.org/10.1016/j.ridd.2024.104797

Santayana de Souza, M., Nobre, G. C., & Valentini, N. C. (2023). Effect of a motor skill-based intervention in the relationship of individual and contextual factors in children with and without developmental coordination disorder from low-income families. *Psychology of Sport and Exercise*, *67*, 102406. https://doi.org/10.1016/j.psychsport.2023.102406

Schwartz, S. P., Northrup, S. R. K., Izadi-Najafabadi, S., & Zwicker, J. G. (2020). Co-op for children with DCD: Goals addressed and strategies used. *Canadian Journal of Occupational Therapy*, *87*(4), 278–286. https://doi.org/10.1177/0008417420941980

Scott, M. W., Wood, G., Holmes, P. S., Marshall, B., Williams, J., & Wright, D. J. (2023). Combined action observation and motor imagery improves learning of activities of daily living in children with developmental coordination disorder. *PLoS ONE*, *18*(5), e0284086. https://doi.org/10.1371/journal.pone.0284086

Sedighi, Z., Arab-Ameri, E., Molanorouzi, K., Ghasemi, A., & Mousavi-Sadati, S. K. (2021). The effect of quiet eye training period manipulation on interceptive skill learning in children with developmental coordination disorder. *Journal of Health System Research*, *17*(2), 133–141. https://doi.org/10.22122/jhsr.v17i2.1418

Sedighi, Z., Arab-Ameri, E., Molanorouzi, K., Ghasemi, A., & Mousavi-Sadati, S. K. (2022). The effect of quiet eye training on performance and kinematics of throwing skill in children with developmental coordination disorder at 6 years old. *Journal of Health System Research*, *17*(4), 311–319. https://doi.org/10.22122/jhsr.v17i4.1439

Seeländer, J., & Fricke, O. (2021). A neurophysiological approach to physiotherapy for the treatment of developmental coordination disorder (DCD) - results of a pilot study. *Physioscience*, *17*(3), 130–136. https://doi.org/10.1055/a-1347-0617

Shahbaz, R., Saemi, E., Doustan, M., Hogg, J. A., & Diekfuss, J. A. (2024). The effect of a visual illusion and self-controlled practice on motor learning in children at risk for developmental coordination disorder. *Scientific Reports*, *14*(1), 12414. https://doi.org/10.1038/s41598-024-63387-z

Shahbazi, S., Rahmani, M., & Heyrani, A. (2016). The effects of sensory-motor integration on balance and reaction time in children with developmental coordination disorder. *Journal of Modern Rehabilitation*, *9*(7), 1–9.

Shahrbanian, S., & Hashemi, A. (2018). The effects of core stabilization training on balance and reaction time in children with developmental coordination disorder. *Research in Sport Management and Motor Behavior*, *8*(16), 83–91.

Shahzad, N., & Jameel, H. T. (2024). Effects of fundamental movement skills training through cooperative learning approach on motivation and confidence of children with developmental coordination disorder to engage in physical activities. *Journal of Applied Research and Multidisciplinary Studies*, *5*(1), 36–41. https://doi.org/10.32350/jarms.51.03

Shank, T. M., & Harron, W. (2015). A retrospective outcomes study examining the effect of interactive metronome on hand function. *Journal of Hand Therapy*, *28*(4), 396–401. https://doi.org/10.1016/j.jht.2015.06.003

Shiozu, H., & Kurasawa, S. (2023). Cognitive orientation to daily occupational performance (co-op) approach as telehealth for a child with developmental coordination disorder: A case report. *Frontiers in Rehabilitation Sciences*, *4*. https://doi.org/10.3389/fresc.2023.1241981

Shire, K. A., Hill, L. J. B., Snapp-Childs, W., Bingham, G. P., Kountouriotis, G. K., Barber, S., & Mon-Williams, M. (2016). Robot guided 'pen skill' training in children with motor difficulties. *PLoS ONE*, *11*(3), 14. https://doi.org/10.1371/journal.pone.0151354

Shoja, M., Mousavi, S. M. K. V., & Ghasemi, A. (2020). Effect of game-centered exercises on the physical self-concept of overweight students with a developmental coordination disorder. *Journal of Health System Research*, *15*(3), 184–191. https://doi.org/10.48305/hsr.2020.15.3.101

Sit, C. H., Yu, J. J., Wong, S. H., Capio, C., & Masters, R. (2019). A school-based physical activity intervention for children with developmental coordination disorder: A randomized controlled trial. *Research in Developmental Disabilities*, *89*, 1–9. https://doi.org/10.1016/j.ridd.2019.03.004

Słowiński, P., Baldemir, H., Wood, G., Alizadehkhaiyat, O., Coyles, G., Vine, S., Williams, G., Tsaneva-Atanasova, K., & Wilson, M. (2019). Gaze training supports self-organization of movement coordination in children with developmental coordination disorder. *Scientific Reports*, *9*(1), 1712. https://doi.org/10.1038/s41598-018-38204-z

Smits-Engelsman, B., Bonney, E., & Ferguson, G. (2020). Motor skill learning in children with and without developmental coordination disorder. *Human Movement Science*, *74*, 102687. https://doi.org/10.1016/j.humov.2020.102687

Smits-Engelsman, B., Bonney, E., & Ferguson, G. (2021). Effects of graded exergames on fitness performance in elementary school children with developmental coordination disorder. *Frontiers in Sports and Active Living*, *3*:653851. https://doi.org/10.3389/fspor.2021.653851

Smits-Engelsman, B. C., Bonney, E., & Jelsma, D. (2023). Task-specificity and transfer of skills in school-aged children with and without developmental coordination disorder. *Research in Developmental Disabilities*, *133*, 104399. https://doi.org/10.1016/j.ridd.2022.104399

Smits-Engelsman, B. C. M., Jelsma, L. D., & Ferguson, G. D. (2017). The effect of exergames on functional strength, anaerobic fitness, balance and agility in children with and without motor coordination difficulties living in low-income communities. *Human Movement Science*, *55*, 327–337. https://doi.org/10.1016/j.humov.2016.07.006

Smits-Engelsman, B. C. M., Jelsma, L. D., Ferguson, G. D., & Geuze, R. H. (2015). Motor learning: An analysis of 100 trials of a ski slalom game in children with and without developmental coordination disorder. *PLoS ONE*, *10*(10), e0140470. https://doi.org/10.1371/journal.pone.0140470

Snapp-Childs, W., Mon-Williams, M., & Bingham, G. P. (2013). A sensorimotor approach to the training of manual actions in children with developmental coordination disorder. *Journal of Child Neurology*, *28*(2), 204–212. https://doi.org/10.1177/0883073812461945

Snapp-Childs, W., Shire, K., Hill, L., Mon-Williams, M., & Bingham, G. P. (2016). Training compliance control yields improved drawing in 5-11year old children with motor difficulties. *Human Movement Science*, *48*, 171–183. https://doi.org/10.1016/j.humov.2016.05.006

Soares, J. C. C., de Moraes, B. L. C., Paz, C. C. S., & Magalhaes, L. D. (2019). Influence of the Microsoft Kinect (R) games on the motor and functional performance of a child with developmental coordination disorder. *Cadernos Brasileiros de Terapia Ocupacional*, *27*(4), 710–717. https://doi.org/10.4322/2526-8910.ctoAO1630

Sos, H., Draxler, A. M., Samwald, M., Graf, I., Nicko, E., Sjencic, A., Haider, M., & Rabl, A. (2019). Das wunstorfer konzept bei einem schulkind mit umschriebener entwicklungsstörung motorischer funktionen (uemf) - eine fallstudie. *Ergoscience*, *14*(3), 90–100. https://doi.org/10.2443/skv-s-2019-54020190301

Straker, L., Howie, E., Smith, A., Jensen, L., Piek, J., & Campbell, A. (2015). A crossover randomised and controlled trial of the impact of active video games on motor coordination and perceptions of physical ability in children at risk of developmental coordination disorder. *Human Movement Science*, *42*, 146–160. https://doi.org/10.1016/j.humov.2015.04.011

Sujatha, B., Alagesan, J., Akahaya, R., Rajameena, R., & Brite Saghaya Rayna, A. (2020). Effect of aerobic exercise training on anxiety in children with developmental coordination disorder. *Biomedicine*, *40*(4), 535–538. https://doi.org/10.51248/.v40i4.336

Sulaiman, M., Baig, M. O., Memon, S. I., Ashraf, N., Butt, P., Afzal, K., & Iqbal, R. (2022). Effects of trampoline exercises in children with developmental coordination disorder: A randomized controlled trial. *Physikalische Medizin Rehabilitationsmedizin Kurortmedizin*, *32*(5), 279–284. https://doi.org/10.1055/a-1755-8655

Tajari, S. N., Gholami, S., Rostami, R., Trabelsi, K., & Taheri, M. (2023). The effect of perceptual-motor exercise on temporal dynamics of cognitive inhibition control in children with developmental coordination disorder. *Mental Health and Physical Activity*, *24:*100495. https://doi.org/10.1016/j.mhpa.2022.100495

Tamplain, P., Sherrod, G. M., Fuchs, C., & Miller, H. L. (2021). Preliminary improvements in dynamic postural control after a group-based intervention program for children with developmental coordination disorder: A brief report. *Developmental Neurorehabilitation*, *24*(1), 63–67. https://doi.org/10.1080/17518423.2020.1819463

Tan, J. L., Siafarikas, A., Rantalainen, T., Hart, N. H., McIntyre, F., Hands, B., & Chivers, P. (2020). Impact of a multimodal exercise program on tibial bone health in adolescents with development coordination disorder: An examination of feasibility and potential efficacy. *Journal of musculoskeletal & neuronal interactions*, *20*(4), 445–471.

Thornton, A., Licari, M., Reid, S., Armstrong, J., Fallows, R., & Elliott, C. (2016). Cognitive orientation to (daily) occupational performance intervention leads to improvements in impairments, activity and participation in children with developmental coordination disorder. *Disability and Rehabilitation*, *38*(10), 979–986. https://doi.org/10.3109/09638288.2015.1070298

Tresser, S. (2012). Case study: Using a novel virtual reality computer game for occupational therapy intervention. *Presence-Virtual and Augmented Reality*, *21*(3), 359–371. https://doi.org/10.1162/PRES_a_00118

Tsai, C.-L., Chang, Y.-K., Chen, F.-C., Hung, T.-M., Pan, C.-Y., & Wang, C.-H. (2014). Effects of cardiorespiratory fitness enhancement on deficits in visuospatial working memory in children with developmental coordination disorder: A cognitive electrophysiological study. *Archives of Clinical Neuropsychology*, *29*(2), 173–185. https://doi.org/10.1093/arclin/act081

Tsai, C.-L., Wang, C.-H., & Tseng, Y.-T. (2012). Effects of exercise intervention on event-related potential and task performance indices of attention networks in children with developmental coordination disorder. *Brain and Cognition*, *79*(1), 12–22. https://doi.org/10.1016/j.bandc.2012.02.004

Tseng, Y.-T., Tsai, C.-L., Wu, T. H., Chen, Y.-W., & Lin, Y.-H. (2023). Table tennis, as a method of sensorimotor training, induces haptic and motor gains in children with a probable developmental coordination disorder. *Motor Control*, *27*(3), 480–497. https://doi.org/10.1123/mc.2021-0123

Valinia, Z., Heirani, A., & Yazdanbakhsh, K. (2017). Effect of eight weeks of perceptual-motor training on working memory in children with developmental coordination disorders. *The Scientific Journal of Rehabilitation Medicine*, *6*(3), 211–220. https://doi.org/10.22037/jrm.2017.1100361

Vilma Alves da Silva, E., Ribeiro Contreira, A., Morgana Felix do Nascimento, E., Capistrano, R., & Silva Beltrame, T. (2013). Motor intervention in schoolchildren with signs of the developmental coordination disorder – DCD. *ConScientiae Saude*, *12*(4), 546–554. https://doi.org/10.5585/ConsSaude.v12n4.4356

Ward, E. J., Hillier, S., Raynor, A., & Petkov, J. (2017). A range of service delivery modes for children with developmental coordination disorder are effective: A randomized controlled trial. *Pediatric Physical Therapy*, *29*(3), 230–236. https://doi.org/10.1097/PEP.0000000000000423

Wilson, P. H., Adams, I. L., Caeyenberghs, K., Thomas, P., Smits-Engelsman, B., & Steenbergen, B. (2016). Motor imagery training enhances motor skill in children with DCD: A replication study. *Research in Developmental Disabilities*, *57*, 54–62. https://doi.org/10.1016/j.ridd.2016.06.014

Wood, G., Miles, C. A. L., Coyles, G., Alizadehkhaiyat, O., Vine, S. J., Vickers, J. N., & Wilson, M. R. (2017). A randomized controlled trial of a group-based gaze training intervention for children with developmental coordination disorder. *PLoS ONE*, *12*(2), e0171782. https://doi.org/10.1371/journal.pone.0171782

Wuang, Y. P., Huang, C. L., & Wu, C. S. (2022). Haptic perception training programs on fine motor control in adolescents with developmental coordination disorder: A preliminary study. *Journal of Clinical Medicine*, *11*(16), 11. https://doi.org/10.3390/jcm11164755

Yasunaga, M., Miyaguchi, H., Ishizuki, C., Kita, Y., & Nakai, A. (2023). Cognitive orientation to daily occupational performance: A randomized controlled trial examining intervention effects on children with developmental coordination disorder traits. *Brain Sciences*, *13*(5). https://doi.org/10.3390/brainsci13050721

Zarei, J., Taheri, H., Sohrabi, M., & Ghasemi, A. (2016). Effect of individual, group and cooperative special practices on the cognitive function of children aged 9-12 years with development coordination disorder. *Iranian Journal of Psychiatry and Clinical Psychology*, *22*(3), 188–199. https://doi.org/10.18869/acadpub.ijpcp.22.3.188

Zwicker, J. G., Rehal, H., Sodhi, S., Karkling, M., Paul, A., Hilliard, M., & Jarus, T. (2015). Effectiveness of a summer camp intervention for children with developmental coordination disorder. *Physical & Occupational Therapy in Pediatrics*, *35*(2), 163–177. https://doi.org/10.3109/01942638.2014.957431
