# Supplementary material for: Interventions for children with developmental coordination disorder: A scoping review
Source: Dev Med Child Neurol. 2026 Feb 26;68(9):1184–94. doi: 10.1111/dmcn.70221 (PMC13436707; doi:10.1111/dmcn.70221)
Supplement: Supplementary file 3 [file DMCN-68-1184-s003.docx]

**Figure S1 Prisma Flowchart**
